# Cinobufagin Directly Targets PDE4D to Disrupt Fibroblast–Dendritic Cell Crosstalk in Atopic Dermatitis

**DOI:** 10.1002/advs.202501670

**Published:** 2025-10-08

**Authors:** Shicong Li, Dihui Xu, Chenyang Zhang, Chunxiu Xiao, Meng Yu, Jiaojiao Wang, Wenyuan Wu, Xiang Lv, Dongping Yuan, Liang Zhang, Min Hong, Jing Zhou, Yang Sun, Hongyue Ma, Yuyu Zhu

**Affiliations:** ^1^ Jiangsu Collaborative Innovation Center of Chinese Medicinal Resources Industrialization and Jiangsu Key Laboratory for High Technology Research of TCM Formulae College of Pharmacy Nanjing University of Chinese Medicine 138 Xianlin Avenue Nanjing 210023 China; ^2^ State Key Laboratory of Pharmaceutical Biotechnology and Department of Rheumatology and Immunology Nanjing Drum Tower Hospital the Affiliated Hospital of Nanjing University Medical School School of Life Sciences Nanjing University Nanjing Jiangsu 210008 China; ^3^ Jiangsu Key Laboratory of New Drug Research and Clinical Pharmacy Xuzhou Medical University Xuzhou 221004 China

**Keywords:** atopic dermatitis, cinobufagin, fibroblast, MIF, PDE4D

## Abstract

Atopic dermatitis (AD) is a chronic inflammatory skin disease driven by immune dysregulation and Th2‐dominant inflammation. This study identifies phosphodiesterase 4D (PDE4D) as a key regulator of AD pathogenesis and a potential therapeutic target. Single‐cell RNA sequencing (scRNA‐seq) revealed activation of the macrophage migration inhibitory factor (MIF) signaling pathway in lesional tissues, with inflammatory fibroblasts mediating MIF‐driven interactions with myeloid cells. Elevated PDE4D expression in lesional tissues suppressed cyclic adenosine monophosphate (cAMP) signaling, promoting inflammation. Cinobufagin, a bufadienolide compound, is identified as a potent PDE4D inhibitor. Compared to other PDE4 inhibitors used in clinical cases, cinobufagin demonstrated superior efficacy in improving AD in mice while effectively regulating the MIF pathway. It directly bound to PDE4D, restored cAMP signaling, suppressed MIF secretion in inflammatory fibroblasts, and disrupted fibroblast–dendritic cell interactions via the cAMP/protein kinase A (PKA)/cAMP‐response element binding protein (CREB) pathway, thereby significantly reducing the clinical and histological features of AD. Notably, PDE4D knockout mice exhibited diminished inflammation, mimicking the effects of cinobufagin and confirming a role for PDE4D in AD progression. These findings establish PDE4D as a critical driver of AD and demonstrate that cinobufagin effectively targets this pathway, offering a promising therapeutic approach for AD and related inflammatory skin disorders.

## Graphical abstract

Single‐cell RNA sequencing revealed that MIF signaling drives inflammatory fibroblastmyeloid cell crosstalk in atopic dermatitis. Cinobufagin, a potent PDE4D inhibitor, suppresses MIF and reduces inflammation by restoring the cAMP/PKA/CREB pathway. Genetic knockout validates PDE4D as a key driver and promising therapeutic target for atopic dermatitis. This figure is created with biorender.com.

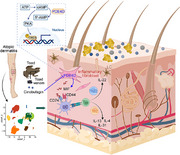

## 1 Introduction

Atopic dermatitis (AD) is a chronic, relapsing inflammatory skin disease that profoundly impacts the quality of life of millions worldwide.^[^ ^1^ ^]^ Characterized by persistent itching, eczematous lesions, and a compromised skin barrier, AD is driven by a complex interplay of genetic predisposition, environmental factors, and immune dysregulation.^[^ ^2^ ^]^ Central to its pathogenesis is an exaggerated immune response, particularly involving T helper 2 cells (Th2), leading to the release of inflammatory cytokines such as interleukin‐4 (IL‐4) and interleukin‐13 (IL‐13) that exacerbate skin inflammation.

Dynamic interactions occur between immune cells, keratinocytes, and fibroblasts within the skin microenvironment.^[^ ^3^ ^]^ Fibroblasts, which have traditionally been viewed as structural components of connective tissue, have now emerged as active participants in the immune response and inflammation.^[^ ^4^ ^]^ In the context of AD, fibroblasts contribute to the inflammatory milieu by secreting a variety of cytokines, chemokines, and growth factors that modulate immune cell recruitment and activation.^[^ ^5^ ^]^ However, the precise mechanisms by which fibroblasts interact with immune cells to exacerbate inflammation in AD remain incompletely understood.

One signaling pathway, the macrophage migration inhibitory factor (MIF) pathway, has been implicated in the progression of various inflammatory diseases, including AD.^[^ ^6^, ^7^ ^]^ MIF, a proinflammatory cytokine secreted by inflammatory fibroblasts, interacts with its receptors CD74 and CD44 to drive immune cell activation and inflammatory responses. Our recent scRNA‐seq studies revealed elevated MIF expression in the lesional tissues of patients with AD, with particularly pronounced expression in inflammatory fibroblasts. The MIF signaling pathway appears to play a central role in fibroblast–myeloid cell interactions by contributing to the recruitment and activation of immune cells, such as dendritic cells (DCs). Despite the importance of MIF, therapeutic strategies targeting its pathway in AD remain largely unexplored.

One interesting response we observed in the lesional tissues of patients with AD is an elevated expression of phosphodiesterase 4D (PDE4D), a member of the PDE4 family of enzymes. The PDE4 enzymes are responsible for the hydrolysis of cyclic adenosine monophosphate (cAMP),^[^ ^8^ ^]^ a key second messenger involved in the regulation of various cellular processes, including immune responses. In patients with AD, the enhanced expression of PDE4D contributes to the suppression of cAMP signaling and the amplification of inflammatory processes. Although previous studies have demonstrated that pharmacological inhibition of PDE4 can reduce inflammation in AD^[^ ^9^, ^10^, ^11^ ^]^; the currently available PDE4 inhibitors, such as roflumilast, are often associated with significant side effects, limiting their clinical utility.^[^ ^12^, ^13^, ^14^ ^]^ Thus, identifying novel and more selective PDE4D‐targeted therapies represents a promising avenue for AD treatment. In this respect, natural compounds could prove to be a valuable source of therapeutic agents for treating inflammatory diseases, such as AD.

In the present study, we explored the use of cinobufagin, a bioactive compound derived from the skin and parotid venom glands of the toad *Bufo bufo gargarizans*,^[^ ^15^, ^16^, ^17^ ^]^ as a potential natural‐source therapeutic for AD. Our results confirm that cinobufagin directly targets PDE4D, thereby modulating cAMP levels and inhibiting the secretion of MIF from fibroblasts. These findings not only highlight the central role of PDE4D in AD pathogenesis but also present cinobufagin as a promising therapeutic candidate for the treatment of AD and related inflammatory skin disorders.

## 2 Results

### 2.1 scRNA‐seq Profiling of Skin from Patients with Atopic Dermatitis

**Figure 1:**
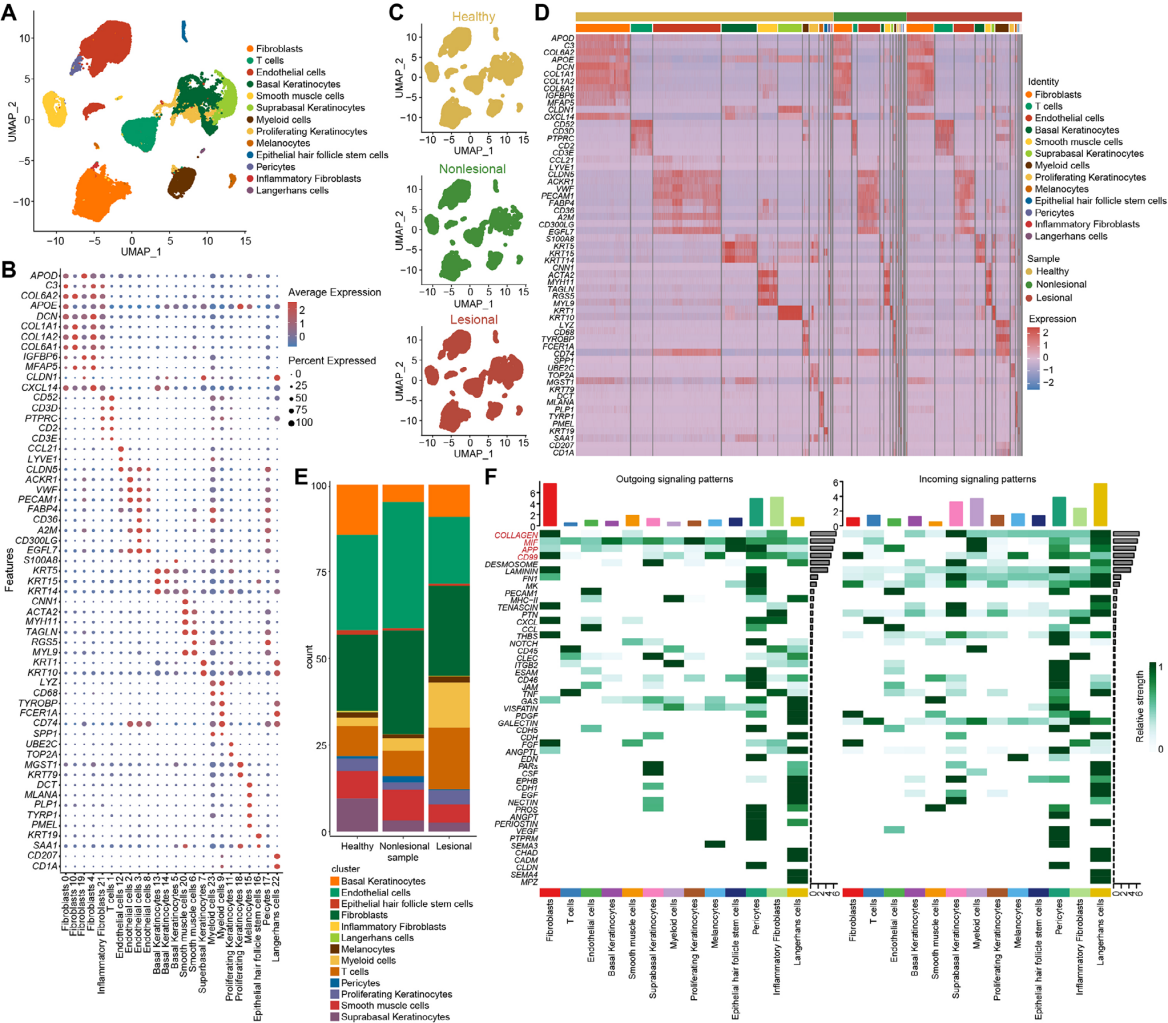
scRNA‐seq profiling of skin from patients with atopic dermatitis. A) UMAP of scRNA‐seq cells recovered from 5 psoriatic patients (4 lesional skin biopsy specimens and 5 non‐lesional skin biopsy specimens) and 7 normal individuals. B) Dot plot for expression of marker genes in skin cell types. C) UMAP of scRNA‐seq cells recovered from healthy, non‐lesional, and lesional groups. D) Heatmap of cluster‐specific genes. E) Proportional map of each cell subpopulation in the skin tissue of healthy, non‐lesional, and lesional groups. F) Strength of outgoing signaling patterns (*left*) and incoming signaling patterns (*right*) by each pathway in each cell subpopulation.

To investigate the skin microenvironment of atopic dermatitis patients, we downloaded single‐cell RNA‐sequencing data (GSE147424) (= GSE147424) from the GENE EXPRESSION OMNIBUS (GEO) database.^[^ ^5^ ^]^ Using this dataset, skin lesions and non‐lesional tissues were obtained from five patients with moderate to severe atopic dermatitis, and normal tissues were obtained from seven healthy individuals. Uniform Manifold Approximation and Projection (UMAP) plots were generated using non‐linear dimensionality reduction with Seurat, and cells were classified into 13 clusters: fibroblasts, inflammatory fibroblasts, T cells, endothelial cells, basal keratinocytes, suprabasal keratinocytes, proliferating keratinocytes, smooth muscle cells, myeloid cells, melanocytes, hair follicle epithelial stem cells, pericytes, and Langerhans cells (**Figure** **1**A). The expression levels of differentially expressed genes in each cell subpopulation are depicted in Figure 1B. We also plotted UMAP maps of normal tissues from healthy individuals and non‐lesional and lesional tissues from patients with AD (Figure 1C). Gene heatmaps for each cell subpopulation demonstrated differentially expressed gene levels, with some subpopulation‐specific genes highlighted (Figure 1D). The proportions of each cell subpopulation in non‐lesional and lesional tissues from patients with AD, as well as in normal tissues from healthy individuals, were analyzed (Figure 1E), revealing significantly increased infiltration by myeloid cells and T cells in lesional tissues compared to both non‐lesional and healthy control tissues. Analysis of the signaling pathways indicated that the COLLAGEN, MIF, APP, and CD99 pathways had the most pronounced interaction intensities (Figure 1F).

### 2.2 The MIF Pathway is Involved in Fibroblast and DC Interactions in the Skin of Patients with Atopic Dermatitis

**Figure 2:**
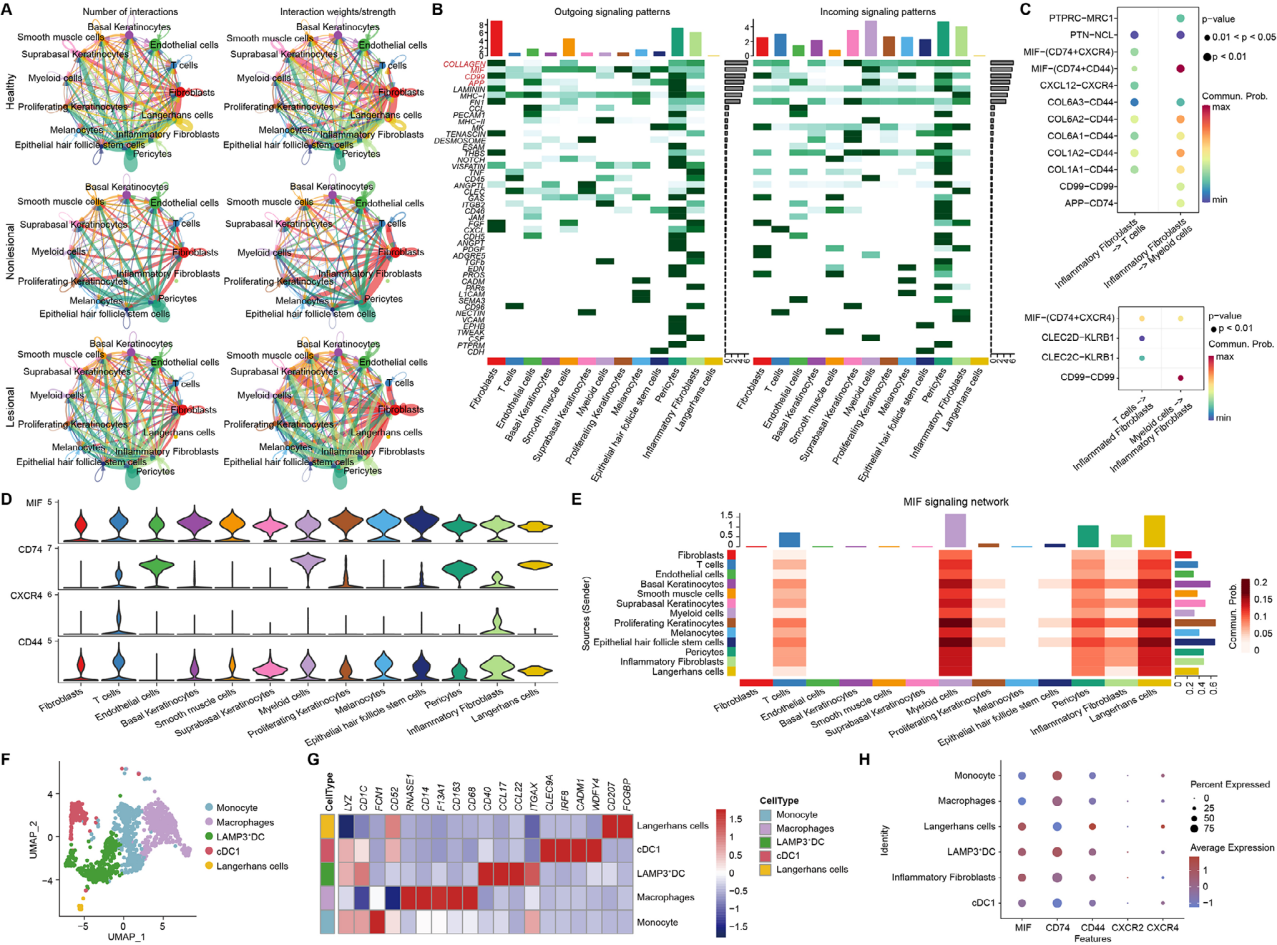
The MIF pathway is involved in fibroblast and DC interactions in the skin of patients with atopic dermatitis. A) Diagram of cell interactions in normal tissues from healthy individuals, non‐lesional and lesional tissues from patients with AD. *left*, number of interactions; *right*: interaction weights/strength. B) Strength of outgoing signaling patterns (*left*) and incoming signaling patterns (*right*) by each pathway in each cell subpopulation of the skin lesions derived from atopic dermatitis patients. C) Bubble diagram demonstrating signaling pathways involved in the regulation of inflammatory fibroblasts on T cells and myeloid cells (*top*) and T cells and myeloid cells on inflammatory fibroblasts (*bottom*) in skin lesion tissues from patients with AD. D) Violin plot demonstrating the expression of MIF signaling pathway‐containing genes in cell subpopulations. E) Heatmap showing visualization of the MIF signaling pathway in cellular interactions. F) UMAP plot of myeloid cells subclusters. G) Heatmap of cluster‐specific genes. H) Bubble diagram demonstrating the expression of MIF signaling pathway‐related genes in myeloid cells.

To examine the role of intercellular interactions in atopic dermatitis disease progression, we analyzed the intercellular interaction network (**Figure** **2**A). Signals from pericytes and fibroblasts were numerous and strong in the normal tissues from healthy individuals and non‐lesional and lesional tissues from patients with AD, with significantly more signals from inflammatory fibroblasts in the lesional tissues of patients with AD. Analysis of the signaling flow patterns in the skin lesion tissues of patients with AD revealed that the COLLAGEN, MIF, CD99, and APP pathways exhibited the most pronounced interaction strengths (Figure 2B). Further analysis of the signaling pathways involved in the regulation of inflammatory fibroblasts to T cells and myeloid cells, as well as T cells and myeloid cells to inflammatory fibroblasts, indicated that the MIF signaling pathway played a critical role in the regulation of inflammatory fibroblasts to myeloid cells (Figure 2C). Our further analysis of the expression levels of relevant genes in the MIF pathway across various cell subpopulations led us to hypothesize that MIF expressed by inflammatory fibroblasts could bind to CD74 and CD44 in myeloid cells, thereby promoting inflammatory responses and exacerbating the disease progression of AD (Figure 2D).

MIF signaling in inflammatory fibroblasts predominantly acts on myeloid and Langerhans cells (Figure 2E). Our genetic analysis of the MIF pathway after further division of myeloid cells into monocytes, macrophages, Langerhans cells, LAMP3^+^ DCs, and cDC1 (Figure 2F,G) revealed that MIF secreted by inflammatory fibroblasts activated downstream signaling pathways mainly by binding to the CD74/CD44 complex of LAMP3^+^ DCs (Figure 2H). The scRNA‐seq data identified the MIF pathway as a central regulator of fibroblast–myeloid cell interactions in AD and a driver of inflammatory responses and disease progression.

### 2.3 Bufadienolides Inhibit the MIF Pathway in Inflammatory Fibroblasts

**Figure 3:**
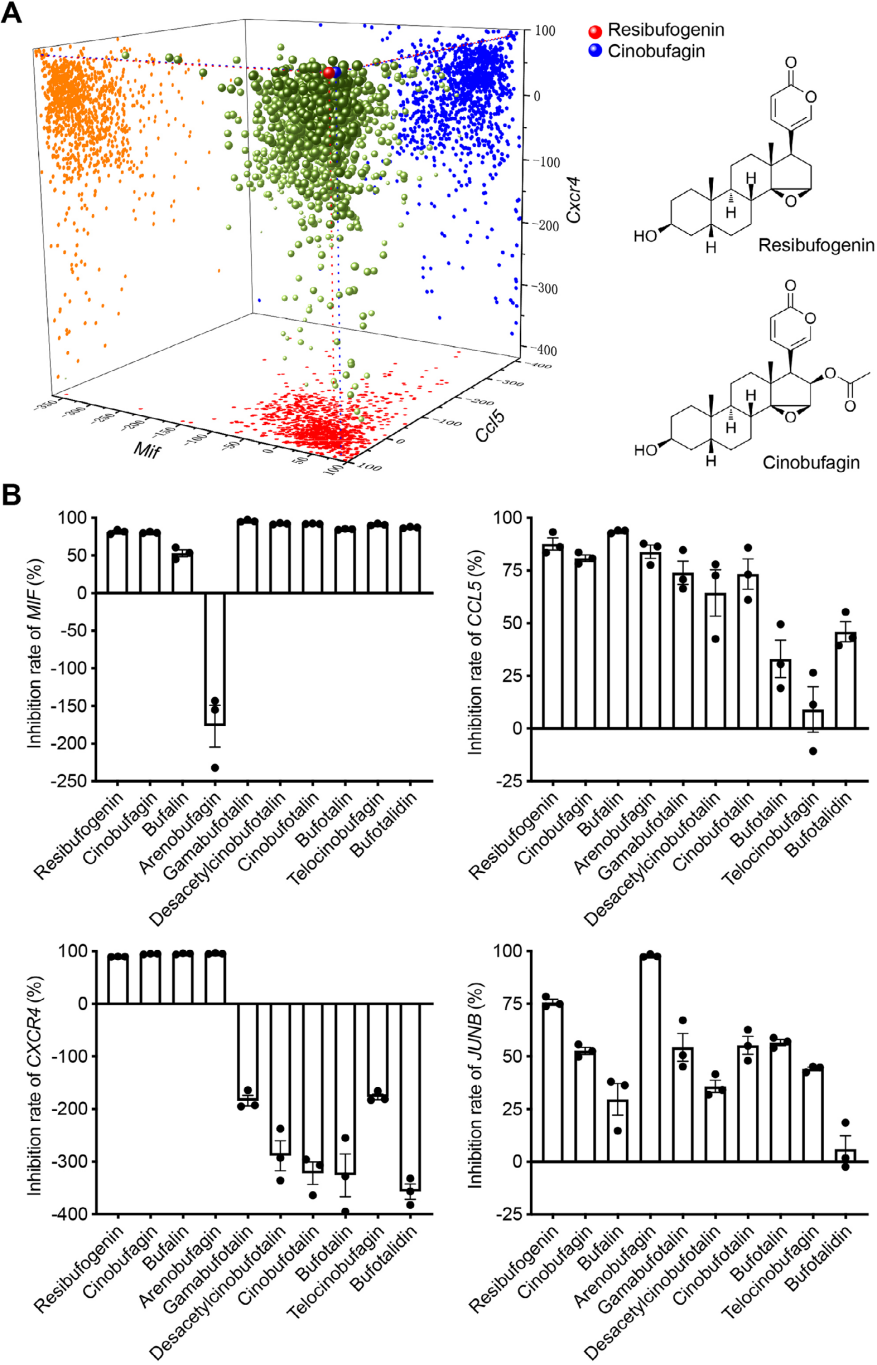
Bufadienolides inhibit the MIF pathway in inflammatory fibroblasts. A) Primary skin fibroblast derived from C57BL/6 mice, untreated or pre‐treated with 1191 natural small‐molecule compounds for 2 h and then stimulated by IL‐4 for 6 h. qPCR analysis of *Mif*, *Ccl5*, and *Cxcr4* mRNA expression. Red ball labelled resibufogenin, and blue ball labelled cinobufagin. B) Human embryonic skin fibroblasts, untreated or pre‐treated with ten individual components of bufadienolides for 2 h and then stimulated by IL‐4 for 6 h. qPCR analysis of *MIF*, *CCL5*, *CXCR4*, and *JUNB* mRNA expression.

The elevated levels of IL‐4 in the microenvironment of skin lesions play a pivotal role in the pathogenesis of atopic dermatitis.^[^ ^18^, ^19^ ^]^ Stimulation of primary mouse skin fibroblasts with IL‐4 to induce their transformation into inflammatory fibroblasts resulted in a significant elevation in the expression of genes related to the MIF pathway (Figure S1, Supporting Information). We screened 1191 natural small‐molecule compounds and identified resibufogenin and cinobufagin as significant inhibitors of the expression of MIF pathway‐related genes, including *Mif*, *Ccl5*, and *Cxcr4*, in inflammatory fibroblasts (**Figure** **3**A). Resibufogenin and cinobufagin are bioactive compounds extracted from toads and are classified as toad sterols, and are the primary active components of toad venom. According to the *Compendium of Materia Medica*, toad‐derived preparations have historically been used to treat various skin conditions, such as chancres, carbuncles, and gangrene.^[^ ^20^, ^21^ ^]^ Based on these findings and historical records, we speculated that bufadienolides may have therapeutic potential for the treatment of AD.

For this reason, we developed a bufadienolides film preparation and treated New Zealand white rabbits infected with *Staphylococcus aureus* (Figure S2, Supporting Information), a primary colonizer and pathogen in AD.^[^ ^22^ ^]^ The bufadienolides treatment reduced skin erythema and decreased epidermal thickening and inflammatory cell infiltration while providing a significant increase in the pain threshold, and an effective reduction in skin charge, indicating the potential for bufadienolides to serve as promising treatments for AD. Our further evaluation of the inhibitory effects of the individual components of bufadienolides on the MIF pathway revealed that the inhibitory effects were significantly stronger for resibufogenin and cinobufagin than for the other components (Figure 3B), highlighting the therapeutic potential of these two compounds as treatments for AD.

### 2.4 Cinobufagin Ameliorates MC‐903‐Induced Atopic Dermatitis in Mice

**Figure 4:**
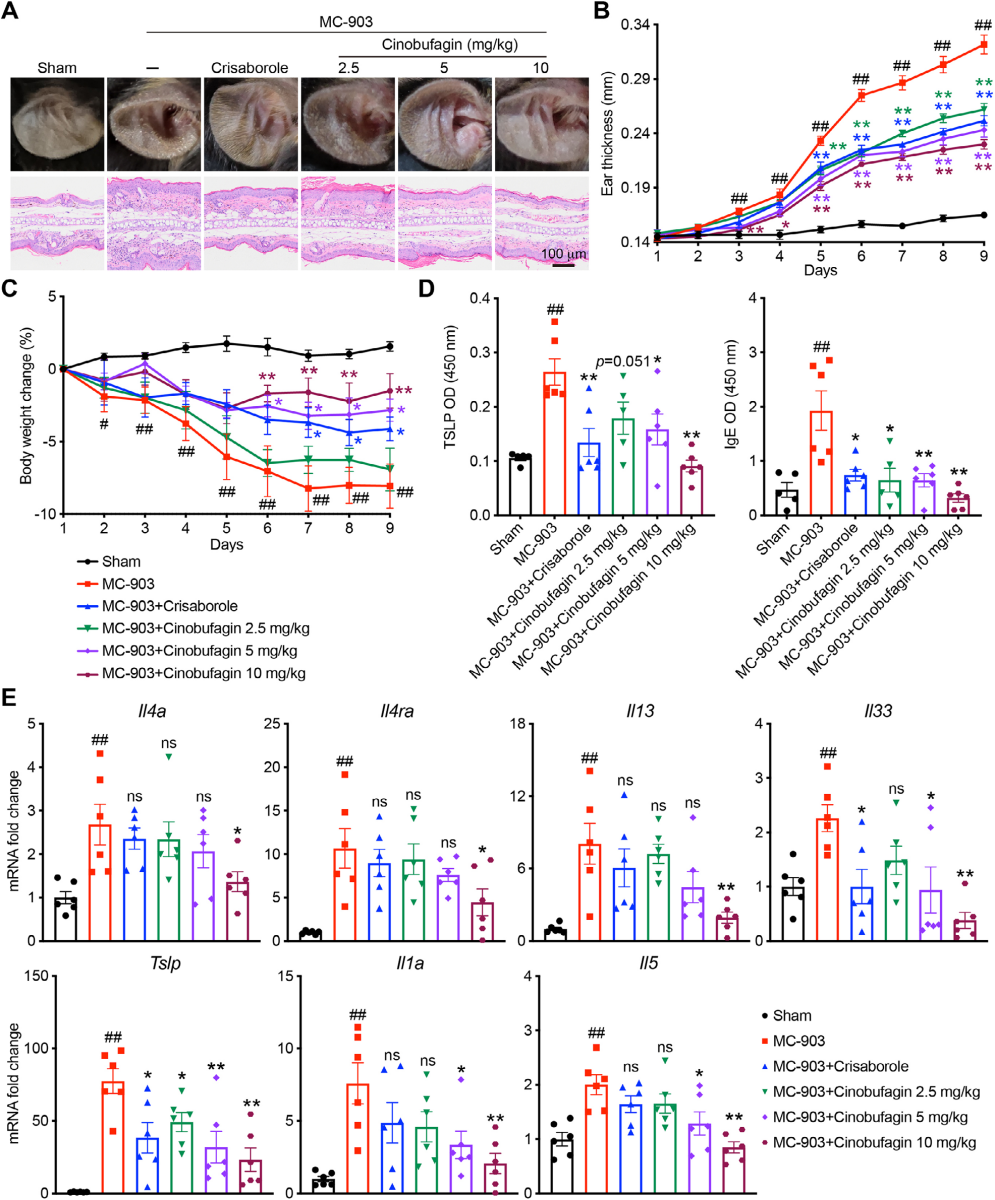
Cinobufagin ameliorates MC‐903‐induced atopic dermatitis in mice. C57BL/6 female mice (*n* = 6/group) were orally administered with the indicated doses of cinobufagin or topically administered 2.4 mg cm^−^^2^ crisaborole once a day for 8 days. A) Phenotypic representation (*top*) and H & E staining (*bottom*) of mouse ears of the indicated groups. Scale bar, 100 µm. B) Thickness of mouse ears of the indicated groups. C) Percent change in body weight of the indicated groups during the disease process. D) ELISA quantification of protein levels of cytokines in mouse serum. E) Quantitative PCR analysis of mRNA encoding Th2 type cytokines in mouse ears. Results were normalized to *Gapdh* expression. Data are represented as mean ± SEM, *n* = 6. *P* values are determined by the Tukey multiple comparison test (B)‐(E). ^#^ *P* < 0.05, ^##^ *P* < 0.01, MC‐903 group versus Sham group; ^*^ *P* < 0.05, ^**^ *P* < 0.01, indicated group versus MC‐903 group.

To evaluate the ameliorative effects of resibufogenin and cinobufagin on AD, we administered resibufogenin and cinobufagin treatments to mice with AD. We replicated the MC‐903‐induced AD model in mice, which exhibits erythema, swelling, and scaling in the ears. Pathological sections revealed epidermal thickening, hyperkeratosis, and infiltration of inflammatory cells in the mouse model group (**Figure** **4**A,B). The model mice also showed a decrease in body weight (Figure 4C), significantly increased levels of thymic stromal lymphopoietin (TSLP) and immunoglobulin E (IgE) in the serum (Figure 4D), and markedly elevated expression of Th2‐type cytokines in the ear tissues (Figure 4E; Figure S3, Supporting Information), reinforcing the Th2‐predominant inflammatory response. The administration of cinobufagin ameliorated the AD‐like damage induced by MC‐903 (Figure 4). Cinobufagin exhibits a favorable safety profile at concentrations relevant to its therapeutic effect for atopic dermatitis (Figure S4, Supporting Information). Resibufogenin also attenuated AD in mice (Figure S5, Supporting Information), although its effect was not as strong as that of cinobufagin. Follow‐up studies will focus on elucidating the mechanism of action of cinobufagin in ameliorating atopic dermatitis.

### 2.5 PDE4D is a Direct Target of Cinobufagin

**Figure 5:**
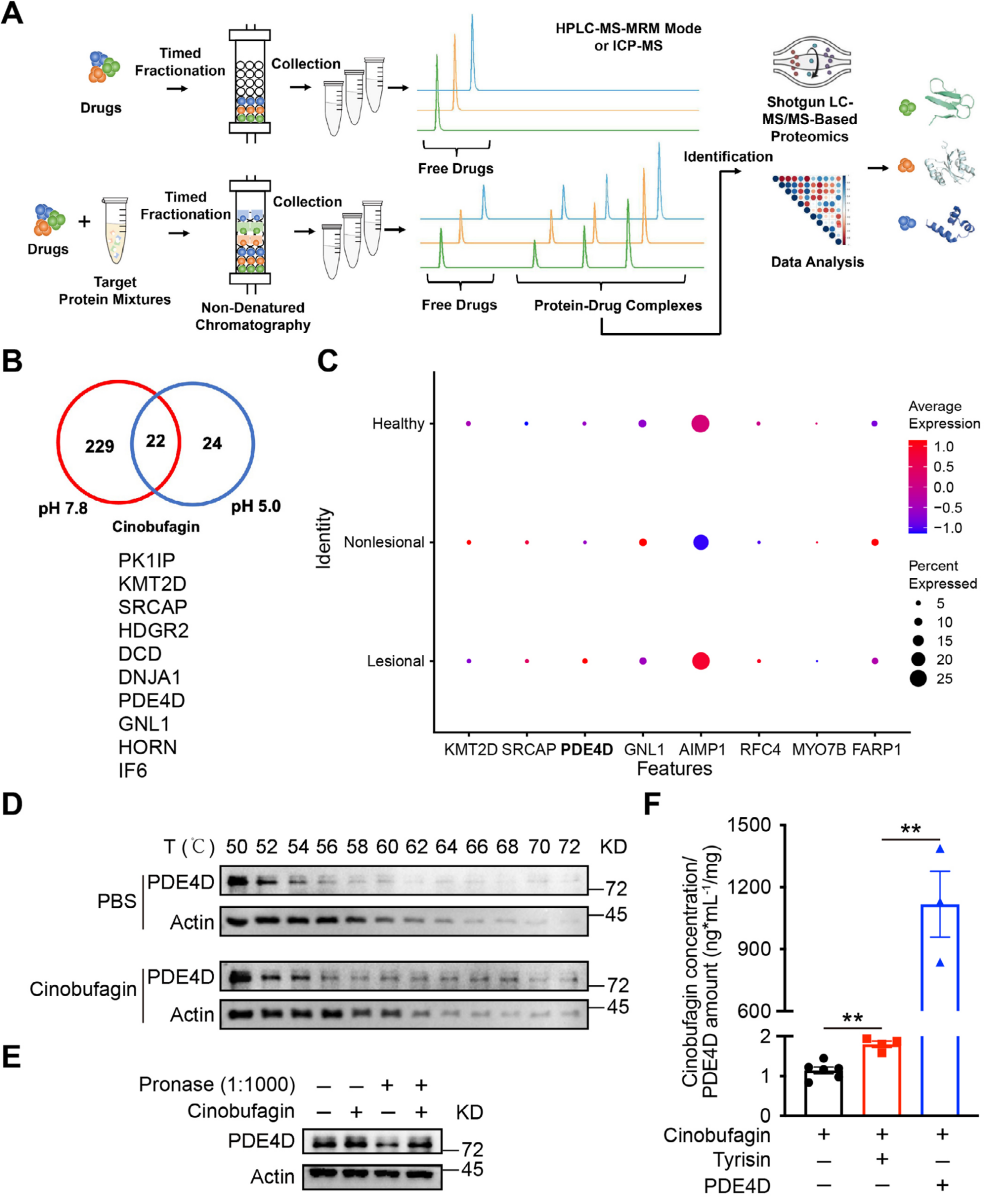
PDE4D is a direct target of cinobufagin. A) Schematic representation of the TICC experiment. B) Venn diagram demonstrating the overlapping potential binding proteins of cinobufagin under pH 7.8 and pH 5.0 elution conditions. C) Expression levels of potential binding proteins of cinobufagin in skin lesions of healthy individuals and atopic dermatitis patients. D) Cells were incubated with cinobufagin or PBS for 2 h, and CETSA analyzed the thermal stabilization of PDE4D protein at different temperatures. E) Cinobufagin enhanced PDE4D resistance to proteases, which was investigated by DARTS. F) Ultrafiltration‐mass spectrometry for drug alone, drug incubated with trypsin, and cinobufagin bound to PDE4D. Data are represented as mean ± SEM, *n* = 3. *P* values are determined by two‐tailed Student's *t*‐test. ^**^ *P* < 0.01.

To further investigate the molecular mechanism by which cinobufagin ameliorates atopic dermatitis, we employed a method for target identification by chromatographic co‐elution (TICC) to identify the target proteins of cinobufagin. Cinobufagin was incubated with cell lysate, followed by gradient fractionation and collection at both pH 7.8 and pH 5.0, to identify the proteins in the cinobufagin binding peaks (**Figure** **5**A). Taking the intersections of the potential binding proteins identified at pH 7.8 and pH 5.0 (Figure S6, Supporting Information), identified 22 potential cinobufagin‐binding proteins (Figure 5B; Table S1, Supporting Information). We then analyzed the expression of the potential target proteins identified by TICC in the skin of patients with AD—both lesional and non‐lesional tissues—as well as in normal skin from healthy individuals by scRNA‐seq, and found that PDE4D expression was significantly elevated in the lesional skin tissues of AD patients (Figure 5C). Cinobufagin also reduced PDE4D protein levels in MC‐903‐induced mice (Figure S7, Supporting Information). These results suggested that PDE4D may be a cinobufagin binding protein.

CCK8 assays showed that low doses of cinobufagin did not affect the proliferation of mouse primary skin fibroblasts (Figure S8, Supporting Information). Further study of the engagement between cinobufagin and PDE4D using cellular thermal shift assays (CETSA) confirmed that cinobufagin significantly enhanced the thermal stability of PDE4D above that of the control group (treated only with PBS), even at high temperatures (Figure 5D). A drug affinity responsive target stability (DARTS) experiment revealed that cinobufagin could potentially stabilize PDE4D (Figure 5E). Ultrafiltration‐mass spectrometry also demonstrated the binding of cinobufagin and PDE4D (Figure 5F), while molecular docking simulations verified that cinobufagin bound to PDE4D (Figure S9, Supporting Information). Taken together, these findings showed that cinobufagin directly binds to PDE4D at both the cellular and molecular levels.

### 2.6 Cinobufagin Targets PDE4D to Regulate MIF Secretion

**Figure 6:**
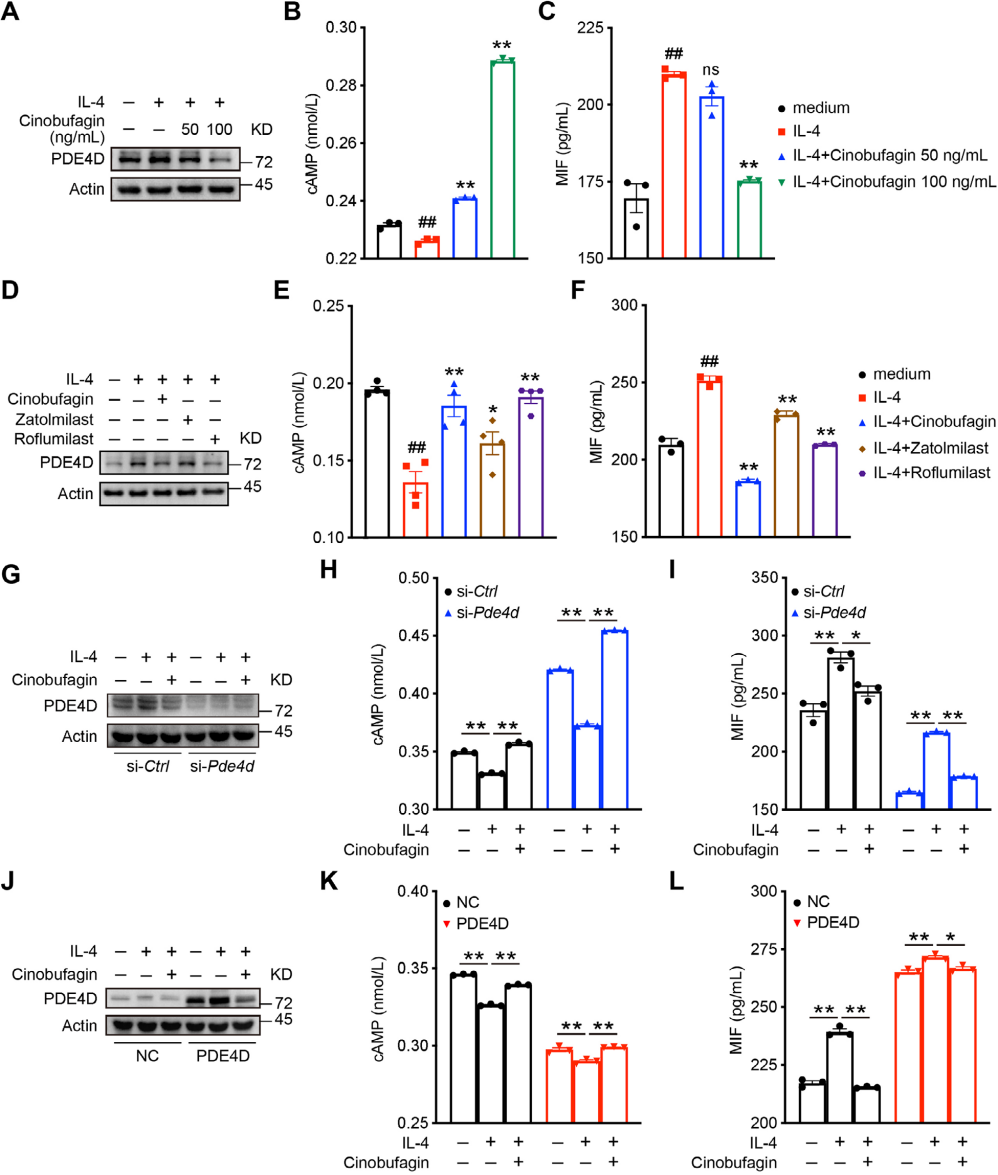
Cinobufagin targets PDE4D to regulate MIF secretion. A–C) Mouse primary skin fibroblasts derived from C57BL/6 mice were stimulated by IL‐4 and treated with or without indicated dose of cinobufagin. Western blot analysis for PDE4D expressions in cell lysates, ELISA for cAMP levels in cell supernatant, and the MIF levels in cell supernatant. D–F) Mouse primary skin fibroblasts derived from C57BL/6 mice were stimulated by IL‐4 and treated with or without PDE4 inhibitors. Western blot analysis for PDE4D expressions in cell lysates, ELISA for cAMP levels in cell supernatant, and the MIF levels in cell supernatant. G–I) PDE4D expression, cAMP levels, MIF levels in mouse primary skin fibroblasts infected with si‐*Ctrl* and si‐*Pde4d* stimulated by IL‐4 and treated with or without cinobufagin. J–L) PDE4D expression, cAMP levels, MIF levels in mouse primary skin fibroblasts infected with NC and PDE4D lentivirus stimulated by IL‐4 and treated with or without cinobufagin. Data are represented as mean ± SEM, *n* = 3. The *P* values are determined by two‐tailed Student's *t*‐test for (B), (C), (E), (F) or Tukey's multiple‐comparison test for (H), (I), (K), (L). ^##^ *P* < 0.01, IL‐4 group versus medium group; ^*^ *P* < 0.05, ^**^ *P* < 0.01, ns, not significant, indicated group versus IL‐4 group.

PDE4D, as a member of the PDE4 family, specifically hydrolyzes cAMP, an important second messenger involved in various physiological processes, including cell proliferation, differentiation, inflammatory response, and neurotransmission.^[^ ^23^, ^24^ ^]^ We investigated whether the targeting of PDE4D by cinobufagin affects MIF secretion through the regulating of cAMP levels by administration of cinobufagin to mouse primary skin inflammatory fibroblasts. The cinobufagin treatment inhibited MIF secretion by suppressing PDE4D expression and upregulating cAMP levels (**Figure** **6**A–C). Compared with zatolmilast, a known PDE4D allosteric inhibitor,^[^ ^25^ ^]^ and roflumilast, a known PDE4 inhibitor,^[^ ^26^ ^]^ cinobufagin had a more pronounced protein inhibition effect on PDE4D (Figure S10, Supporting Information). Cinobufagin also triggered a greater elevation of cAMP levels and a greater reduction in MIF levels (Figure 6D–F). Therefore, the targeted inhibition of PDE4D by cinobufagin increased cAMP levels and decreased MIF levels, as further confirmed in our animal experiments (Figure S11, Supporting Information).

Our subsequent establishment of fibroblasts showing PDE4D knockdown and overexpression confirmed the results observed for inhibition of PDE4D, as knockdown of PDE4D increased cAMP levels and decreased MIF secretion (Figure 6G–I), while overexpression of PDE4D had the opposite effect (Figure 6J–L). Taken together, our findings suggested that PDE4D can positively regulate MIF expression.

### 2.7 Cinobufagin Disrupts Fibroblast–DC Interactions via cAMP/PKA/CREB Signaling Pathway

**Figure 7:**
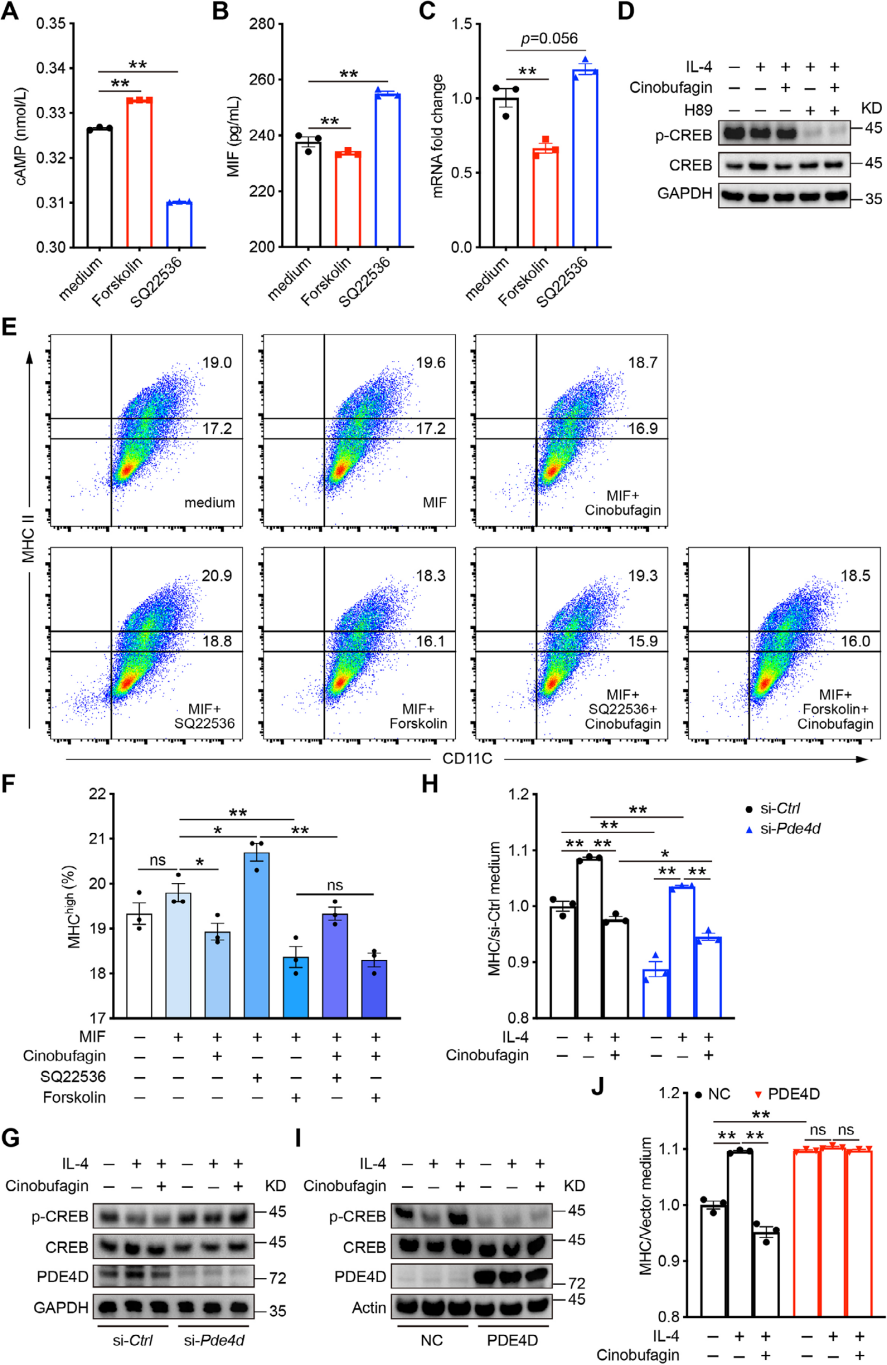
Cinobufagin disrupts fibroblast–DC interactions via cAMP/PKA/CREB signaling pathway. A–C) Mouse primary skin fibroblasts derived from C57BL/6 mice were treated with cAMP agonist (forskolin) and inhibitor (SQ22536), and ELISA quantification of protein levels of cAMP and MIF, *Mif* mRNA expression in cell supernatant. D) Immunoblotting staining of mouse primary skin fibroblasts derived from C57BL/6 mice were stimulated by IL‐4 and treated with or without cinobufagin or H‐89. E) Primary mouse skin fibroblasts were stimulated with MIF, treated with cinobufagin or forskolin, or SQ22536, or a combination of these, and then co‐cultured with BMDCs for 12 h. The BMDCs were collected, and flow cytometry was performed to detect the expression of CD11C and MHC II. F) Frequencies of MHC II^high^ BMDCs gated on BMDCs in (E). G, I) Phosphorylation levels of CREB in knockdown and overexpression of PDE4D mouse primary skin fibroblasts. H,J) Knockdown and overexpression of PDE4D mouse primary skin fibroblasts were stimulated by IL‐4 and treated with or without cinobufagin, and then co‐cultured with BMDCs for 12 h. The BMDCs were collected, and flow cytometry was performed to detect the expression of CD11C and MHC II. Proportion of MHC BMDCs in each group versus the medium group. Data are represented as mean ± SEM, *n* = 3. The *P* values are determined by two‐tailed Student's *t*‐test for (A‐C), (F) or Tukey's multiple‐comparison test for (H), (J). ^*^ *P* < 0.05, ^**^ *P* < 0.01, ns, not significant.

To confirm whether cAMP levels affect MIF expression, we used cAMP agonist forskolin^[^ ^27^ ^]^ and inhibitor SQ22536^[^ ^28^ ^]^ to stimulate fibroblasts (**Figure** **7**A), and found that MIF levels could be reduced by forskolin and enhanced by SQ22536 treatments (Figure 7B,C). Recognizing that the key transcription factor downstream of the cAMP signaling pathway is the CREB, we next investigated the role of the cAMP/PKA/CREB pathway in the regulation of MIF‐mediated inflammatory responses modulated by PDE4D. CREB plays a critical role in inflammatory diseases by transducing signals through the cAMP‐PKA‐CREB axis to regulate gene transcription and expression.^[^ ^29^, ^30^ ^]^ When fibroblasts were treated with cinobufagin, they exhibited increased levels of phosphorylated CREB (p‐CREB), indicating activation of the cAMP/PKA/CREB pathway. However, the group treated with both cinobufagin and the PKA inhibitor H‐89^[^ ^31^ ^]^ showed suppression of p‐CREB levels increases (Figure 7D; Figure S12, Supporting Information), suggesting that cinobufagin activates the cAMP/PKA/CREB signaling pathway to regulate MIF secretion.

We further explored the interaction between fibroblasts and DCs, by culturing fibroblasts with mouse bone marrow–derived DCs (BMDCs) (Figure S13, Supporting Information). Administration of cinobufagin or forskolin, a cAMP agonist, to fibroblasts inhibited the expression of major histocompatibility complex II (MHC II) in DCs. MHC II is a core molecule essential for DCs function, as it determines their ability to capture, process, and present antigens, as well as to regulate immune responses. Therefore, MHC II expression can serve as a functional marker of DC activity.^[^ ^32^, ^33^ ^]^ Conversely, the administration of the cAMP inhibitor SQ22536 promoted DC function, but this effect was reversed by cinobufagin (Figure 7E,F), suggesting that cinobufagin disrupts fibroblast–DC interactions by modulating cAMP signaling.

We further elucidate the role of PDE4D in this process by constructing fibroblasts with PDE4D knockdown or overexpression. Knocking down PDE4D increased CREB phosphorylation levels and subsequently inhibited DC function (Figure 7G,H), whereas overexpression of PDE4D reduced CREB phosphorylation levels and enhanced DC function (Figure 7I,J). Collectively, these findings indicate that cinobufagin, by targeting PDE4D, regulates the cAMP/PKA/CREB signaling pathway in fibroblasts, thereby inhibiting fibroblast–DC interactions and DC‐mediated immune responses.

### 2.8 PDE4D Knockout Reduces the Severity of Atopic Dermatitis in Mice

**Figure 8:**
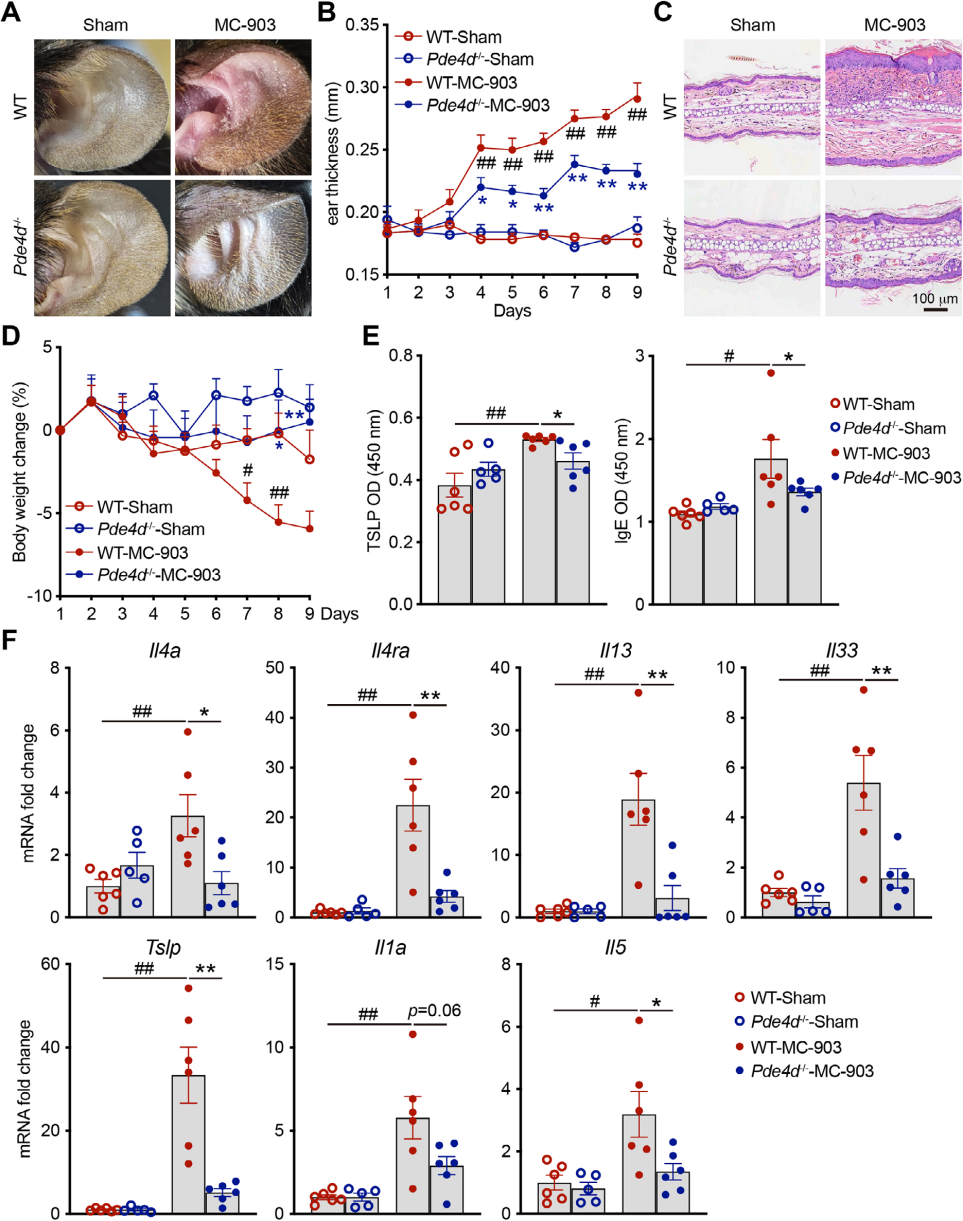
PDE4D knockout reduces the severity of atopic dermatitis in mice. Wild‐type (*n* = 6) and *Pde4d* ^−/‐^ male and female mice (*n* = 5–6) were treated with MC‐903 for nine days. A) Phenotypic representation of mouse ears of the indicated groups. B) Thickness of mouse ears of the indicated groups during the disease process. C) H & E staining of mouse ears of the indicated groups. Scale bar, 100 µm. D) Percent change in body weight of the indicated groups during the disease process. E) ELISA quantification of TSLP and IgE levels in mouse serum. F) Quantitative PCR analysis of mRNA encoding Th2 type cytokines in mouse ears. Results were normalized to *Gapdh* expression. Data are represented as mean ± SEM, *n* = 5–6. *P* values are determined by Tukey's multiple‐comparison test (B), (D)‐(F). ^#^ *P* < 0.05, ^##^ *P* < 0.01, WT‐MC903 group versus WT‐Sham group; ^*^ *P* < 0.05, ^**^ *P* < 0.01, *Pde4d* ^−/−^‐MC‐903 group versus WT‐MC‐903 group.

To investigate the role of PDE4D in atopic dermatitis, we constructed PDE4D knockout (*Pde4d* ^−/−^) mice, and confirmed a pretty efficiency of *Pde4d* deletion (Figure S14, Supporting Information). We administered MC‐903 to the ears of the wild‐type (WT) and *Pde4d* ^−/‐^ mice. Compared to wild‐type mice, the *Pde4d* ^−/‐^ mice exhibited significantly reduced disease severity. Key clinical features, including ear redness and scaling (**Figure** **8**A), and ear thickness (Figure 8B), were markedly alleviated in the *Pde4d* ^−/‐^ mice. Histological analysis revealed decreased inflammatory cell infiltration and reduced ear epidermal thickness in the PDE4D‐deficient mice (Figure 8C). Additionally, the systemic indicators of disease severity, such as body weight loss (Figure 8D) and serum levels of TSLP and IgE (Figure 8E), were significantly lower in the *Pde4d* ^−/‐^ mice than in the WT mice. The expression of Th2‐type cytokines was also downregulated in the ear tissues of the PDE4D knockout mice (Figure 8F; Figure S15, Supporting Information).

These results suggested that PDE4D plays a critical role in the development and progression of AD and that targeted inhibition of PDE4D could represent another approach for the treatment of AD. Notably, the administration of cinobufagin to the MC‐903‐induced PDE4D knockout mice did not further improve their morbidity (Figure S16, Supporting Information). These findings suggested that the therapeutic effect of cinobufagin is highly dependent on the presence of PDE4D and support the idea that PDE4D is its main pharmacological target.

## 3 Discussion

Atopic dermatitis is a multifaceted inflammatory skin disease with a complex pathogenesis that involves immune dysregulation, epidermal barrier dysfunction, and microbial colonization. Recent advances have highlighted the role of specific immune pathways, including Th2 cytokines (IL‐4, IL‐13) and innate immune responses, in driving AD disease progression.^[^ ^34^, ^35^ ^]^ Moreover, scRNA‐seq studies have provided unparalleled insights into the cellular and molecular heterogeneity of AD, particularly regarding the interactions between structural and immune cells in lesional tissues.^[^ ^5^, ^36^, ^37^ ^]^ Our findings presented here build on these advancements by identifying PDE4D as a critical regulator of AD pathogenesis and a promising therapeutic target.

Consistent with the growing body of research demonstrating the importance of fibroblasts in skin inflammation,^[^ ^38^, ^39^, ^40^ ^]^ our study revealed that inflammatory fibroblasts are central mediators of MIF signaling in AD. Elevated MIF expression in lesional tissues, particularly in inflammatory fibroblasts, drives pathogenic interactions with myeloid cells, such as LAMP3^+^ dendritic cells, through CD74/CD44 signaling. These findings align with previous work linking fibroblast dysfunction to immune dysregulation in chronic inflammatory diseases^[^ ^5^ ^]^ and further highlight the critical role of fibroblast–immune cell crosstalk in AD.

Our discovery of PDE4D as a key driver of inflammation in AD expands the growing interest in phosphodiesterases as therapeutic targets in dermatological and systemic inflammatory diseases. PDE4 inhibitors, such as roflumilast and apremilast, have shown efficacy in controlling inflammatory conditions, such as psoriasis and AD; however, their clinical potential is often limited by off‐target effects and systemic toxicity. In this study, we demonstrated that PDE4D is significantly upregulated in the lesional tissues of AD patients, where it serves to suppress cAMP signaling and amplifying inflammatory responses. These findings are consistent with previous research identifying PDE4 as a regulator of inflammation,^[^ ^41^, ^42^, ^43^ ^]^ but our work uniquely pinpoints PDE4D as a critical isoform driving AD pathogenesis. The most important result was that the genetic deletion of PDE4D in mice recapitulated the anti‐inflammatory effects observed with pharmacological inhibition, confirming the central role of PDE4D in AD disease progression.

Cinobufagin, a bufadienolide derived from toad venom, was identified as a potent PDE4D inhibitor. Global medical research has increasingly focused on natural compounds as sources of novel therapeutics, with bufadienolides being explored for their anti‐inflammatory^[^ ^44^, ^45^ ^]^ and anti‐cancer^[^ ^46^, ^47^ ^]^ properties. Our study is the first to demonstrate that cinobufagin directly binds to PDE4D. Notably, in our PDE4D knockout mice, the therapeutic effect of cinobufagin on AD was abolished, suggesting that the therapeutic efficacy of cinobufagin highly depends on the presence of PDE4D and indicating that PDE4D may be the primary, if not exclusive, functional target of cinobufagin. By inhibiting PDE4D, cinobufagin restored cAMP signaling, suppressed MIF secretion in inflammatory fibroblasts, and disrupted fibroblast–DC interactions via the cAMP/PKA/CREB pathway. Compared to currently identified PDE4 inhibitors now used in clinical practice, such as crisaborole, cinobufagin demonstrated superior efficacy in improving AD symptoms in mice.

A critical consideration for any bufadienolide is its potential for systemic toxicity. To address this, we performed a comprehensive safety assessment. Our therapeutic in vitro concentration (100 ng mL^−1^) demonstrated no cytotoxic effects in skin fibroblasts, keratinocytes, macrophages, or, crucially, in H9c2 cardiomyocytes (Figure S8, Supporting Information). These findings were substantiated in vivo, where daily oral administration of a high dose (10 mg kg^−1^) for eight days induced no signs of systemic toxicity (Figure S4, Supporting Information). Notably, while minor electrocardiogram variations were observed, key cardiac function parameters remained statistically unchanged, confirming the absence of significant cardiotoxicity at this therapeutic dose. Taken together, these data establish a clear therapeutic window for cinobufagin, positioning it as a viable clinical candidate for AD.

While PDE4 inhibitors such as apremilast have gained FDA approval for psoriasis, and while ongoing trials are evaluating their use in AD, the adverse effects, such as gastrointestinal toxicity, associated with these inhibitors remain a significant limitation hindering their clinical use.^[^ ^48^, ^49^ ^]^ Our results suggest that cinobufagin, with its dual ability to inhibit PDE4D and suppress MIF‐mediated inflammation, as a promising therapeutic candidate. Critically, our study was designed to validate cinobufagin as a proof‐of‐concept for a novel oral, systemic therapy. This approach addresses the significant unmet clinical need for effective oral treatments in patients with moderate‐to‐severe AD, for whom topical therapies are often insufficient. The choice of oral administration was further guided by the physicochemical properties of cinobufagin, which predict poor dermal permeability, making an oral route more viable for achieving therapeutic concentrations. Therefore, our in vivo model was not intended to be a direct replacement for the topical control but rather to establish the efficacy of a different therapeutic modality. Although comparing disparate administration routes is complex, the systemic exposure achieved with oral cinobufagin provides a potent therapeutic effect. While future research could explore advanced topical formulations to overcome cinobufagin's permeability challenges—thereby localizing its effects and minimizing systemic exposure—our current findings establish it as a compelling lead compound for a much‐needed oral treatment for widespread inflammatory skin disease.

Our study aligns with global efforts to develop PDE4 inhibitors with improved safety profiles and enhanced tissue specificity, thereby positioning cinobufagin as a promising therapeutic candidate for AD. Nonetheless, several challenges remain. First, while our mouse model recapitulates many aspects of AD, it cannot fully capture the chronic, relapsing–remitting nature of the disease in humans. Second, although cinobufagin demonstrated efficacy in suppressing MIF signaling and restoring cAMP levels, its broader effects on other signaling pathways and cell types in AD require further investigation. Finally, as with any natural compound, the scalability of cinobufagin production and its pharmacokinetics and safety profile must be rigorously evaluated in preclinical and clinical trials.

In conclusion, our study identifies PDE4D as a critical driver of AD pathogenesis and highlights cinobufagin as a novel therapeutic agent capable of targeting this pathway. By disrupting fibroblast–immune cell interactions and modulating cAMP signaling, cinobufagin effectively attenuates the inflammation associated with AD. Our findings not only advance the understanding of AD pathogenesis, but they also align with global efforts to develop more targeted, safe, and effective treatments for chronic inflammatory skin diseases. Our future research will focus on optimizing cinobufagin formulations and validating their efficacy in clinical settings to fully realize the therapeutic potential of this effective PDE4D inhibitor.

## 4 Experimental Section

### Animals

C57BL/6 mice were purchased from GemPharmatech Co., Ltd (Nanjing, China). PDE4D knockout mice were generated using the CRISPR/Cas9 system. CRISPR/Cas9 system and donor were microinjected into the fertilized eggs of C57BL/6JGpt mice. Fertilized eggs were transplanted to obtain positive F0 mice, which were confirmed by PCR and sequencing. A stable F1 generation mouse model was obtained by mating positive F0 generation mice with C57BL/6JGpt mice. These mice were bred and maintained in specific pathogen‐free conditions at GemPharmatech Co., Ltd (Nanjing, China) and the Experimental Animal Center at Nanjing University of Chinese Medicine (Approval Number: 202405A071). The mice used in the study were of a C57BL/6 background, age‐ and sex‐matched, and 8–10 weeks old. Animals were housed in a controlled environment with a 12‐h light–dark cycle at 22 °C, with ad libitum access to standard laboratory chow and water.

### Single‐Cell RNA Sequencing (scRNA‐seq)

The single‐cell RNA sequencing (scRNA‐seq) data were obtained from the GEO database under accession number GSE147424. Data analysis was performed using the R package Seurat (v4.0.0). Cell type identities were consistent with the annotations provided in the supplementary files from the GEO database. Quality control criteria were applied to retain cells with mitochondrial genes < 10% and nFeature_RNA > 200. Data preprocessing and normalization were performed using the NormalizeData function, while dimensionality reduction was achieved with the RunPCA and RunTSNE functions for principal component analysis and non‐linear dimensional reduction, respectively. Target gene expression density was visualized using the plot_density function from the R package Nebulosa (v4.0.0). Additionally, cell–cell interactions among different cell types were inferred using CellChat (v1.6.1).

### MC‐903‐Induced Atopic Dermatitis‐Like Mouse Model

Eight‐ to ten‐week‐old C57BL/6 wild‐type or transgenic mice were used to establish this model. Mice received a daily topical application of MC‐903 (2 nmol L^−1^ dissolved in 20 µL ethanol) on the dorsal side of the right ear for nine consecutive days. Control mice were treated with 20 µL of ethanol on the same area. Ear thickness and body weight were measured daily. Mice were sacrificed one day after the final treatment to collect serum and ear tissues. Serum samples were used for ELISA, while ear tissues were processed for hematoxylin and eosin (H&E) staining, immunofluorescence, and qPCR analysis.

### Histological Analysis

Mice ear tissues were rinsed with PBS, fixed in 4% formaldehyde overnight, and embedded in paraffin. Tissue sections (5 µm thick) were stained with hematoxylin and eosin (H&E) following standard procedures.

For immunofluorescence, paraffin‐embedded ear sections were deparaffinized, rehydrated, and subjected to antigen retrieval using sodium citrate. Sections were blocked with 5% goat serum and incubated overnight at 4 °C with primary antibodies, including anti‐PDE4D (Proteintech, catalog 12918‐1‐AP), anti‐Vimentin (Proteintech, catalog 60330‐1‐Ig), and anti‐p‐CREB (CST, catalog 9198T), all diluted 1:100. After three washes with 1× PBST, sections were incubated with Alexa Fluor 594‐conjugated goat anti‐rabbit IgG (H + L) (1:500; Invitrogen, catalog A‐11034) and Alexa Fluor 488‐conjugated goat anti‐mouse IgG (H + L) (1:500; Invitrogen, catalog A‐32723) for 2 h at room temperature in the dark. Nuclei were counterstained with DAPI. All the cells were imaged by an inverted confocal microscope (Carl Zeiss).

### Quantitative PCR (qPCR)

Total RNA was extracted from mouse skin and ear tissues, as well as from primary mouse skin fibroblasts and peritoneal macrophages, using the RNA Isolater Total RNA Extraction Reagent (Vazyme Biotech Co., Ltd, China) according to the manufacturer's instructions. Reverse transcription was performed using 1 µg of total RNA to synthesize single‐stranded cDNA. Real‐time PCR was conducted using the AceQ Universal SYBR qPCR Master Mix (Vazyme Biotech Co., Ltd, China) on a CFX 100 cycler (Bio‐Rad, Hercules, CA). Primer sequences are listed in Table S2 (Supporting Information). The amplification protocol consisted of an initial denaturation at 95 °C for 2.5 min, followed by 40 cycles of 95 °C for 15 s and 60 °C for 30 s. Dissociation curve analysis was performed at the end of the amplification to confirm specificity. Gene expression levels were normalized to *Gapdh* RNA expression.

### Enzyme‐Linked Immuno Sorbent  Assay (ELISA)

Serum samples were assayed by ELISA with mouse TSLP (88‐7490‐22‐2, Invitrogen), mouse IgE (88‐50460‐22‐2, Invitrogen), mouse cAMP (YFXEM00846, Yfxbio Biotech. Co. Ltd. (Nanjing, China)), or MIF (ELK2466, ELK Biotechnology) kits according to the manufacturer's instructions.

### Target Identification by Chromatographic Co‐elution (TICC)

The cinobufagin was mixed and incubated with cell lysate, reduced by adding dithiothreitol (DTT), then modified by iodoacetamide (IAA), enzyme‐digested with trypsin, and desalted by OASIS HLB solid phase extraction column. Subsequently, Nano LC‐MS/MS analysis was performed using a Q Exactive Plus mass spectrometer in combination with EASY‐nLC II, and database search and protein identification were performed by PEAKS software.

### Cellular Thermal Shift Assay (CETSA)

CETSA was performed as previously described.^[^ ^50^ ^]^ Primary mouse skin fibroblasts were treated with either 100 ng mL^−1^ cinobufagin or an equivalent volume of PBS (control). After 2 h, cells were collected, washed twice with PBS, and resuspended in 650 µL PBS. The suspensions were aliquoted into 12 PCR tubes (50 µL each) and subjected to a temperature gradient (50–72 °C in 2 °C increments) using a PCR thermocycler. Each sample was heated for 3 min, cooled at room temperature for 3 min, and then placed on ice. After heating, samples underwent three freeze‐thaw cycles (−80 °C overnight, thawed at room temperature, then re‐frozen for 2 h). Following processing, samples were centrifuged at 20000 × *g* for 20 min at 4 °C. Supernatants were mixed with 6× loading buffer, heated at 95 °C for 5 min, and analyzed by SDS‐PAGE.

### Drug Affinity Responsive Target Stability (DARTS)

The cell lysate was evenly divided into four PCR tubes: two were treated with 100 ng mL^−1^ cinobufagin, and two were treated with an equivalent volume of DMSO as controls. After overnight incubation, samples were digested with streptavidin protease for 10 min. The reaction was terminated by adding loading buffer, and the samples were analyzed by immunoblotting.

### Affinity Ultrafiltration

Cinobufagin (1 µg mL^−1^) was incubated with vehicle, bovine serum albumin (BSA), or PDE4D for 30 min at 4 °C and room temperature, respectively. The mixture was transferred into a 10 kDa ultrafiltration chamber (Millipore) and centrifuged at 8000 rpm at 4 °C for 30 min. The retentate was washed with buffer (10 mmol L^−1^ Tris‐HCl, 0.1 mmol L^−1^ DTT) and centrifuged again under the same conditions. The ultrafiltrate was collected, reduced with 5 mmol L^−1^ DTT, and alkylated with 15 mmol L^−1^ IAA, followed by trypsin digestion. Peptides were analyzed using an EASY‐nLC II system (Thermo Fisher Scientific) coupled to a Q Exactive Plus mass spectrometer (Thermo Fisher Scientific). Desalted peptides were auto‐sampled onto a self‐packed column (20 cm × 75 µm i.d., 3 µm C18 beads, 120 Å) and eluted over 90 min with a linear gradient of 5–90% acetonitrile in 0.1% formic acid at a flow rate of 300 nL min^−1^. Raw mass spectrometry data were processed using Peaks Studio 8.5.

### Molecular Docking

Genes PDE4D were selected and confirmed the relevant information through UniProt database (https://www.uniprot.org), the receptor protein 3D structure of PDE4D in PDB format was obtained and downloaded from the RCSB PDB database (https://www.rcsb.org). Ligand compound of cinobufagin was obtained through SciFinder (https://scifinder‐n.cas.org) to obtain a 3D structure file in MOL format. Preparation for docking protein receptors and ligand compounds using Schrödinger software (Maestro 11.5, Schrödinger 2018), complete docking through the Maestro sector. After the docking was completed, a score table with docking score, glide gscore, and glide emodel as the main evaluation indicators was obtained for result analysis. The protein structure was synthesized with the compound structure, and the synthesized structure was visualized using PyMol 2.5.4 for analysis.

### Western Blotting

Cell samples were lysed using Cell Lysis Buffer for Western and IP (Beyotime, China) supplemented with protease and phosphatase inhibitors (MCE). Protein concentrations were determined with the Bradford assay (HyClone‐Pierce). Proteins were separated by SDS‐PAGE and transferred onto polyvinylidene difluoride membranes. The membranes were incubated with primary antibodies overnight at 4 °C, followed by incubation with horseradish peroxidase‐conjugated secondary antibodies. Protein detection was performed using the LumiGLO chemiluminescent substrate system. The following primary antibodies were used: anti‐PDE4D (1:1000; Proteintech, catalog 12918‐1‐AP), anti‐p‐CREB (1:1000; CST, catalog 9198T), anti‐CREB (1:1000; CST, catalog 9197T), anti‐*β*‐Actin (1:2000; Abmart, catalog M20011), and anti‐GAPDH (1:2000; Abmart, catalog M20006).

### RNA Interference

Small interfering RNA (siRNA) targeting *Pde4d* was synthesized by GENERAL BIOL: sense: GCCAGUGUGAUAUACACGGAGAUTT, antisense: AUCUCCGUGUAUAUCACACUGGCTT. The siRNA molecules were transfected using a Hieff Trans in vitro siRNA/miRNA Transfection Reagent (Yeasen Biotechnology (Shanghai) Co. Ltd.).

### Lentiviral Transduction

Lentiviruses targeting PDE4D and a negative control (NC) were purchased from Shanghai Obio Technology Co., Ltd. (Shanghai, China). Fibroblasts were transfected with either NC or PDE4D lentivirus and incubated for 72 h. Infected cells were then selected with puromycin (10 µg mL^−1^; Sigma–Aldrich) for 14 days. Protein levels and gene expression were subsequently analyzed by immunoblotting and qPCR, respectively.

### Cell Co‐Culture

Bone marrow cells were extracted from mouse bone marrow and stimulated with granulocyte‐macrophage colony‐stimulating factor (GM‐CSF, 20 ng mL^−1^; Absin, catalog abs05214) and interleukin‐4 (IL‐4, 10 ng mL^−1^; TargetMol, catalog TMPY‐02558) to induce differentiation into dendritic cells. The dendritic cell‐containing culture medium was then added to fibroblasts with PDE4D knockdown, overexpression, or cinobufagin treatment to establish a fibroblast–dendritic cell co‐culture system.

### Statistics

Statistical analysis was performed using GraphPad Prism 9.0. Data are presented as the mean ± SEM. Statistical significance was analyzed using a two‐tailed Student's *t*‐test or Tukey's multiple‐comparison test. A *P* value < 0.05 was considered statistically significant.

## Conflict of Interest

The authors declare no conflict of interest.

## Author Contributions

S. L. and D.X. contributed equally to this work. Y.Z., H.Y., Y.S., and J.Z. conceived this project and designed the study. S.L. and D.X. performed the experiments. Y.Z., C.Z., C.X., M.Y., and J.W. analyzed the data. W.W., X. L., D.Y., L.Z., and M.H. gave methodological support and conceptual advice. Y.S. and Y.Z. wrote the manuscript. All authors discussed the results and commented on the manuscript.

## Supporting information



Supporting Information

## Data Availability

Research data are not shared.
