# Supplementary material for: Cinobufagin Directly Targets PDE4D to Disrupt Fibroblast–Dendritic Cell Crosstalk in Atopic Dermatitis
Source: Adv Sci (Weinh). 2025 Oct 8;12(48):e01670. doi: 10.1002/advs.202501670 (PMC12752632; doi:10.1002/advs.202501670)
Supplement: Supplementary file 1 — Supporting Information [file ADVS-12-e01670-s001.docx]

**Supplementary Information for**

**Cinobufagin Directly Targets PDE4D to Disrupt Fibroblast–Dendritic Cell Crosstalk in Atopic Dermatitis**

**This PDF file includes:**

**Supplementary Figures 1 to 16**

**Supplementary Tables 1 and 2**

**
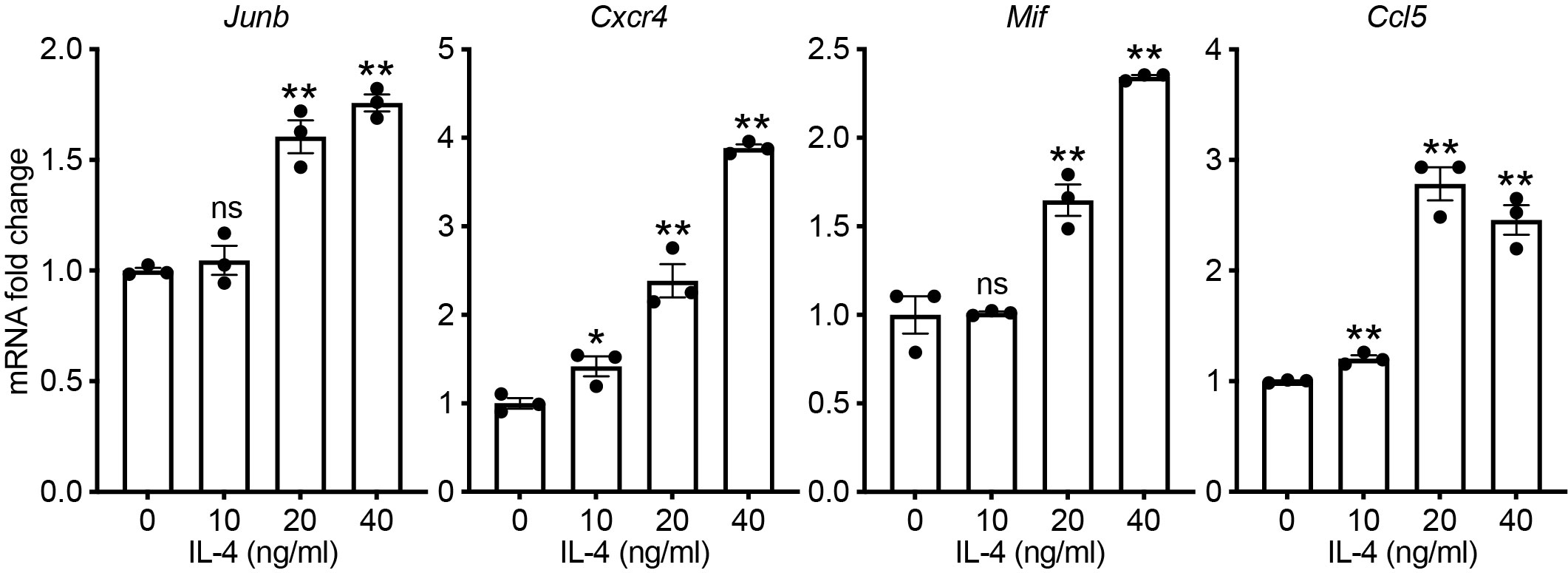
**

**Figure S1.** Modeling inflammatory fibroblasts. Quantitative PCR analysis of mRNA expression of critical encoding genes of the MIF pathway in mouse primary skin fibroblasts stimulated by the indicated doses of IL-4 for 6 h. Results were normalized to *Gapdh* expression. Data are represented as mean ± SEM, *n* = 3. *P* values are determined by two-tailed unpaired Student’s *t*-test. **P* < 0.05, ***P* < 0.01, ns, not significant, indicated group vs. 0 group.


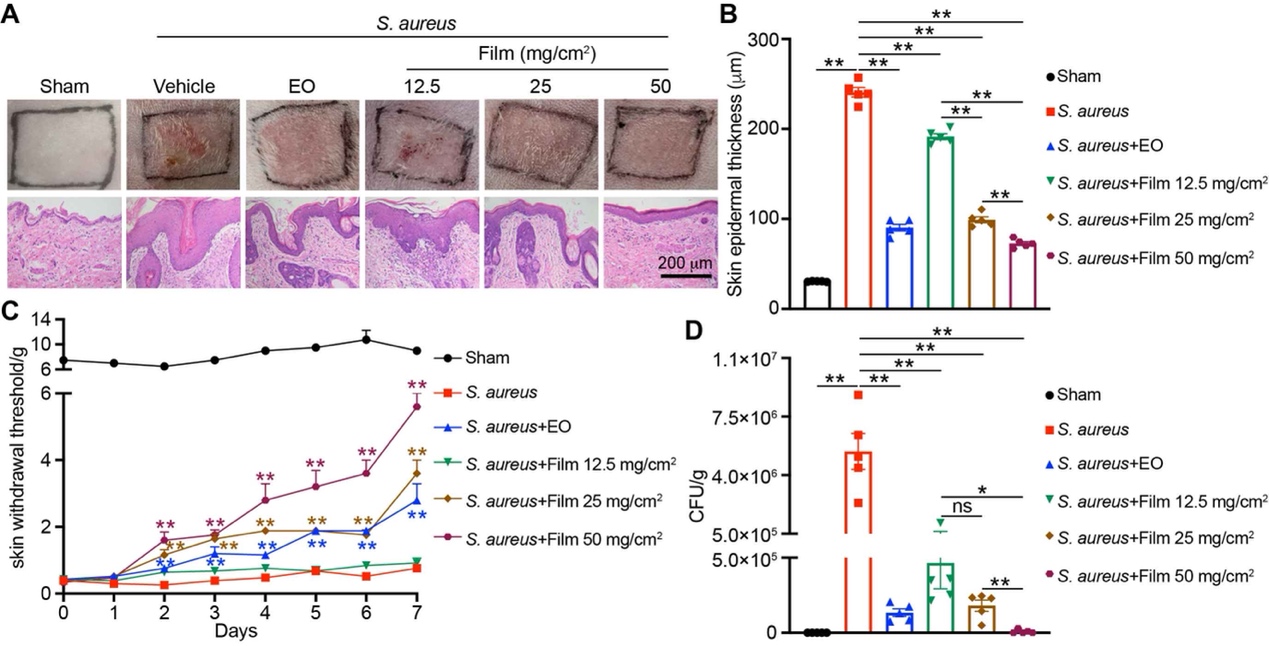


**Figure S2.** Bufadienolides film ameliorates *Staphylococcus aureus* infection in New Zealand white rabbit. New Zealand white rabbit topical application of indicated dose of bufadienolides film or erythromycin ointment (EO) for 7 days. A) Phenotypic representation (*top*) and H&E staining (*bottom*) of the dorsal skin of New Zealand white rabbit infected with *Staphylococcus aureus*. Scale bar, 200 μm. B) Skin epidermal thickness statistics for each group in (A). C) Skin withdrawal thresholds on the back of the New Zealand white rabbit. D) Colony forming unit (CFU) on the back of the New Zealand white rabbit. Data are represented as mean ± SEM, *n* = 5. *P* values are determined by Tukey multiple-comparison test (B)-(D). **P* < 0.05, ***P* < 0.01, ns, not significant.


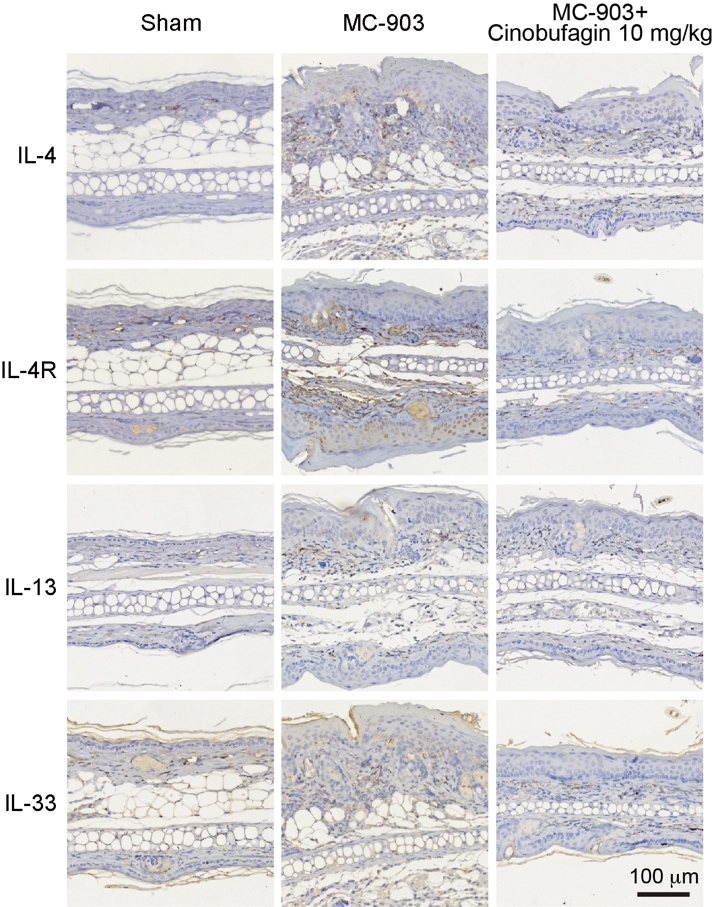


**Figure S3.** Cinobufagin reduces the expression of Th2-type cytokines in the ears of MC-903-induced AD mice. Detection of IL-4, IL-4R, IL-13, and IL-33 in ear tissues of Sham, MC-903-induced, and cinobufagin-treated C57BL/6 mice. Scale bar, 100 μm.


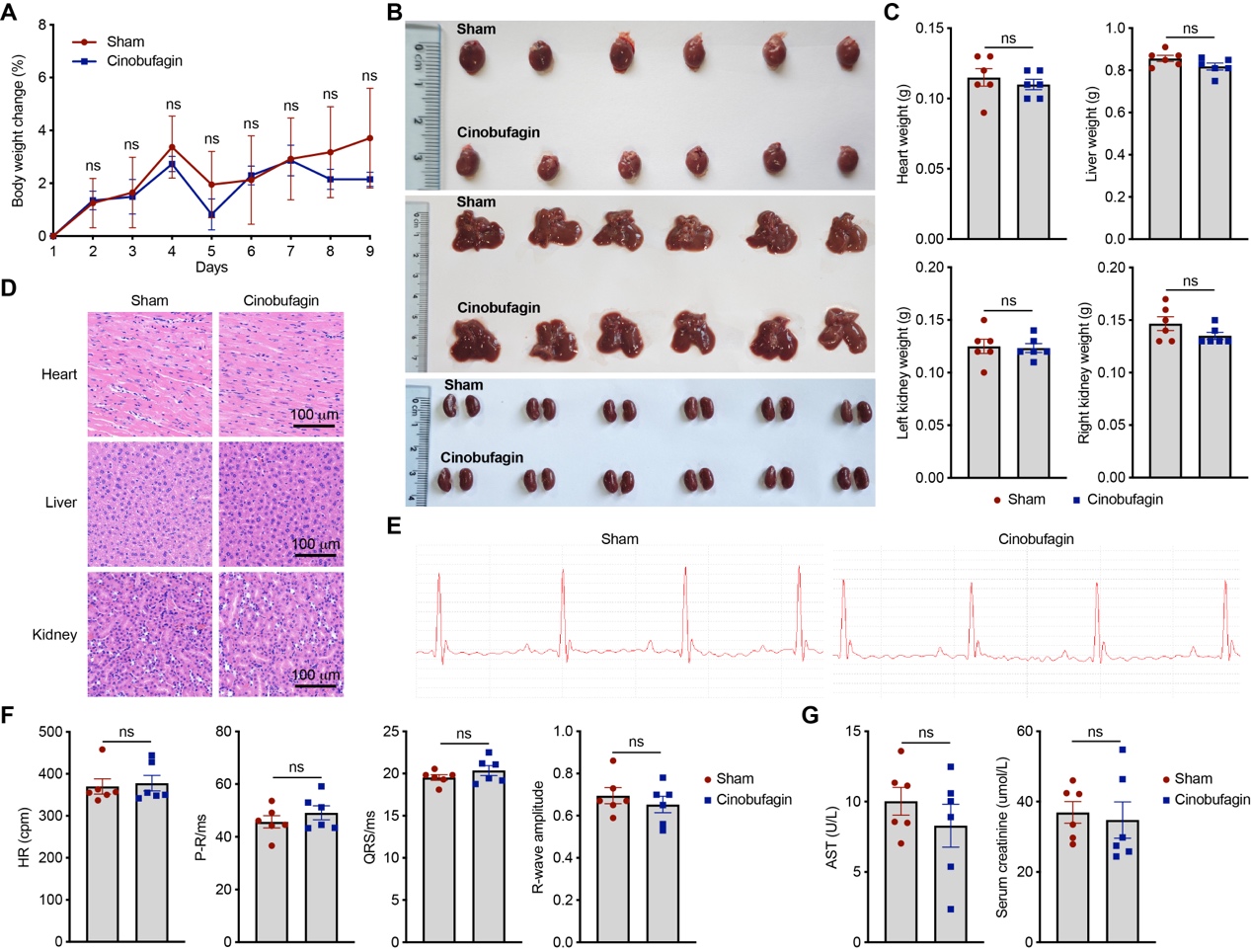


**Figure S4.** Systemic safety and toxicity assessment of oral cinobufagin administration in mice. C57BL/6 male mice were orally administered either vehicle (Sham) or cinobufagin (10 mg/kg) daily for eight days. A) Percentage change in body weight monitored daily throughout the treatment period. B) Representative images showing the gross morphology of hearts, livers, and kidneys harvested at the end of the study. C) Quantitative analysis of heart, liver, left kidney, and right kidney weights. D) H&E staining of mouse heart, liver, and kidney. Scale bar, 100 μm. E) Representative electrocardiogram (ECG) traces from individual mice in both the Sham and Cinobufagin groups. F) Quantification of key ECG parameters, including heart rate (HR), P-R interval, QRS duration, and R-wave amplitude. G) Serum levels of aspartate aminotransferase (AST) and creatinine were measured to assess liver and kidney function, respectively. Data are presented as mean ± SEM, *n*=6. *P* values are determined by two-tailed unpaired Student’s *t*-test. ns, not significant.


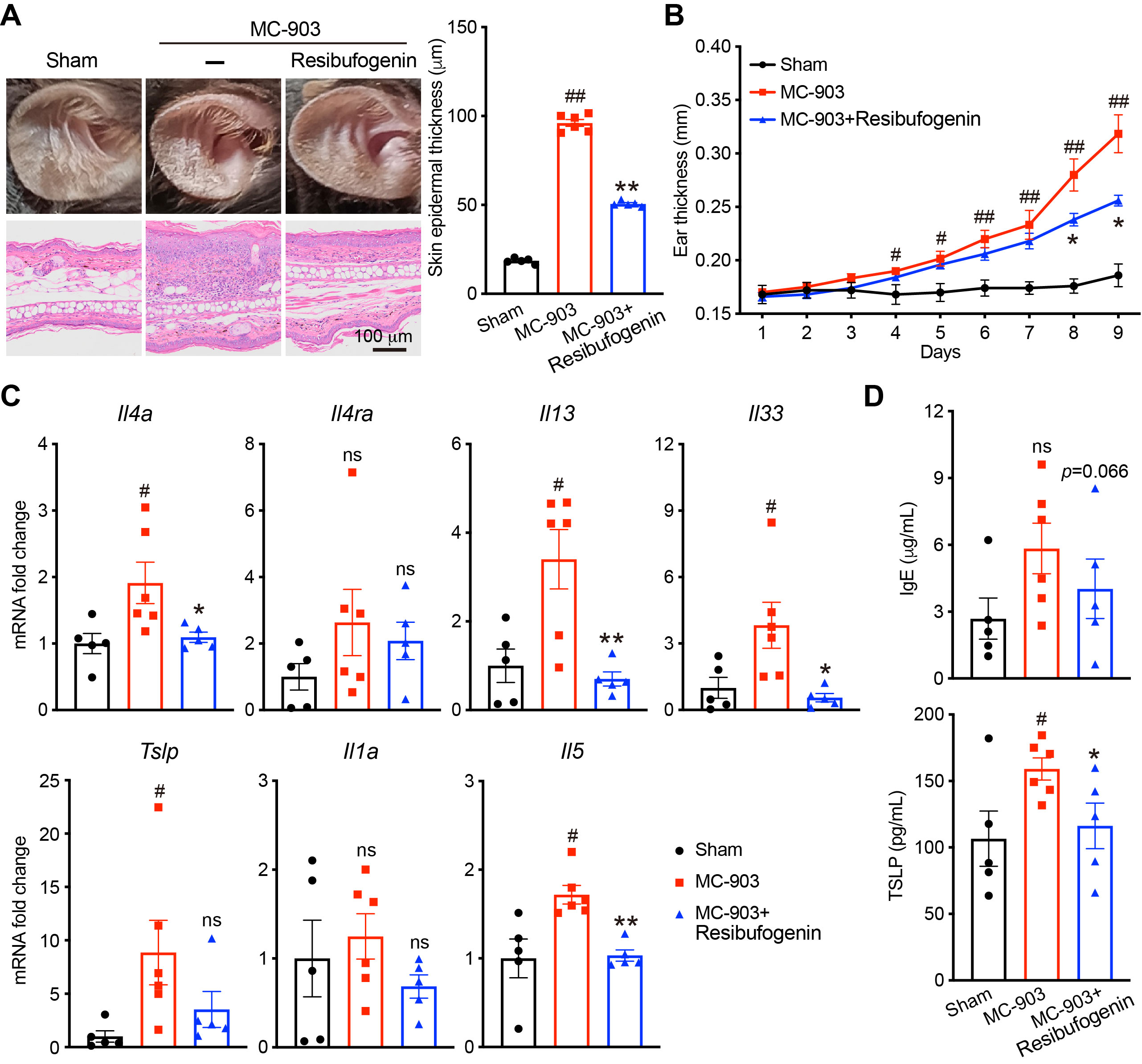


**Figure S5.** Resibufogenin certainly ameliorates MC-903-induced atopic dermatitis in mice. C57BL/6 female mice (*n* = 6/group) were orally administered with resibufogenin or vehicle for 8 days. A) Phenotypic representation (*top*) and H&E staining (*bottom*) of mouse ears of the indicated groups. Skin epidermal thickness statistics for each group on the *right*. Scale bar, 100 μm. B) Thickness of mouse ears of the indicated groups. C) Quantitative PCR analysis of mRNA encoding Th2 type cytokines in mouse ears. Results were normalized to *Gapdh* expression. D) ELISA quantification of protein levels of cytokines in mouse serum. Data are represented as mean ± SEM, *n* = 6. *P* values are determined by two-tailed unpaired Student’s *t*-test. ^#^*P* < 0.05, ^##^*P* < 0.01, ns, not significant, MC-903 group vs. Sham group; **P* < 0.05, ***P* < 0.01, ns, not significant, resibufogenin group vs. MC-903 group.


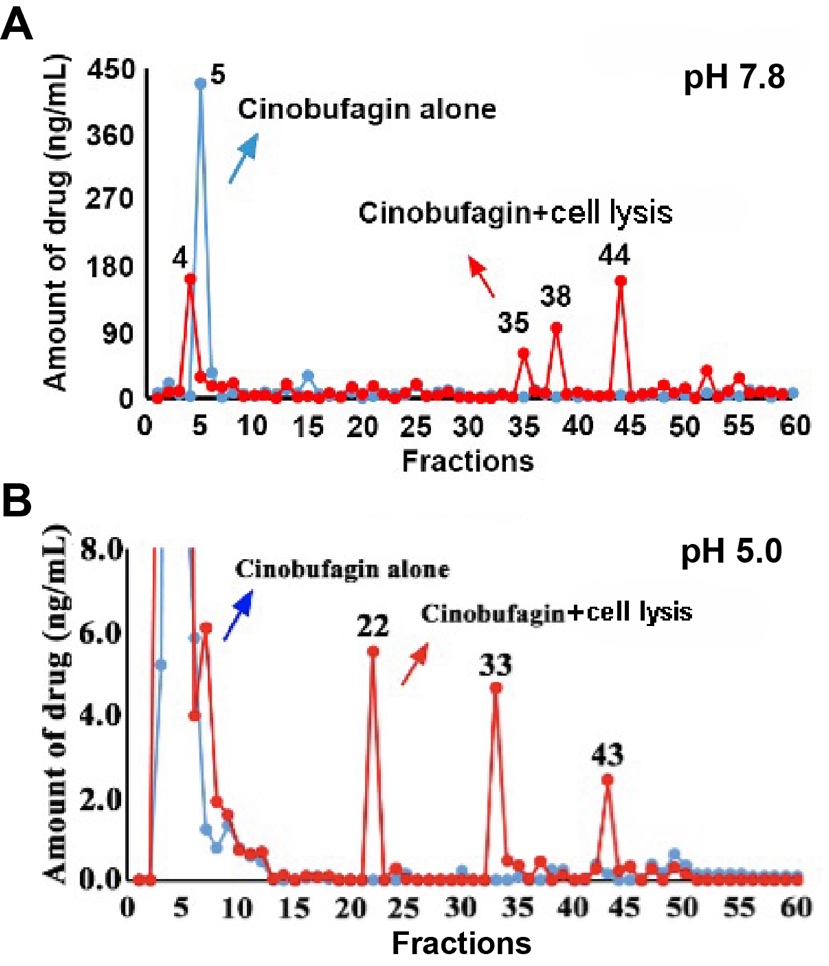


**Figure S6.** Cinobufagin binding peaks. Binding peaks of cinobufagin under pH 7.8 (A) and pH 5.0 (B) elution conditions.


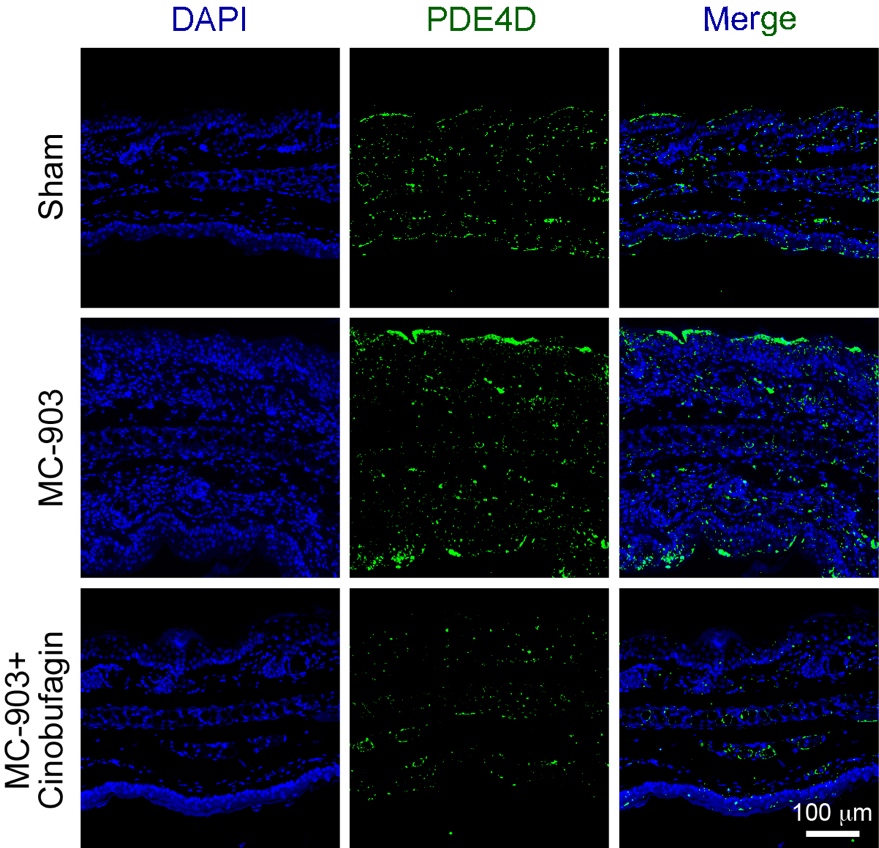


**Figure S7.** Cinobufagin inhibits the expression of PDE4D in the ears of MC-903-induced AD mice. Ear skin sections from Sham, MC-903-induced, and cinobufagin-treated C57BL/6 mice were immune-stained for PDE4D. Scale bar: 100 μm.


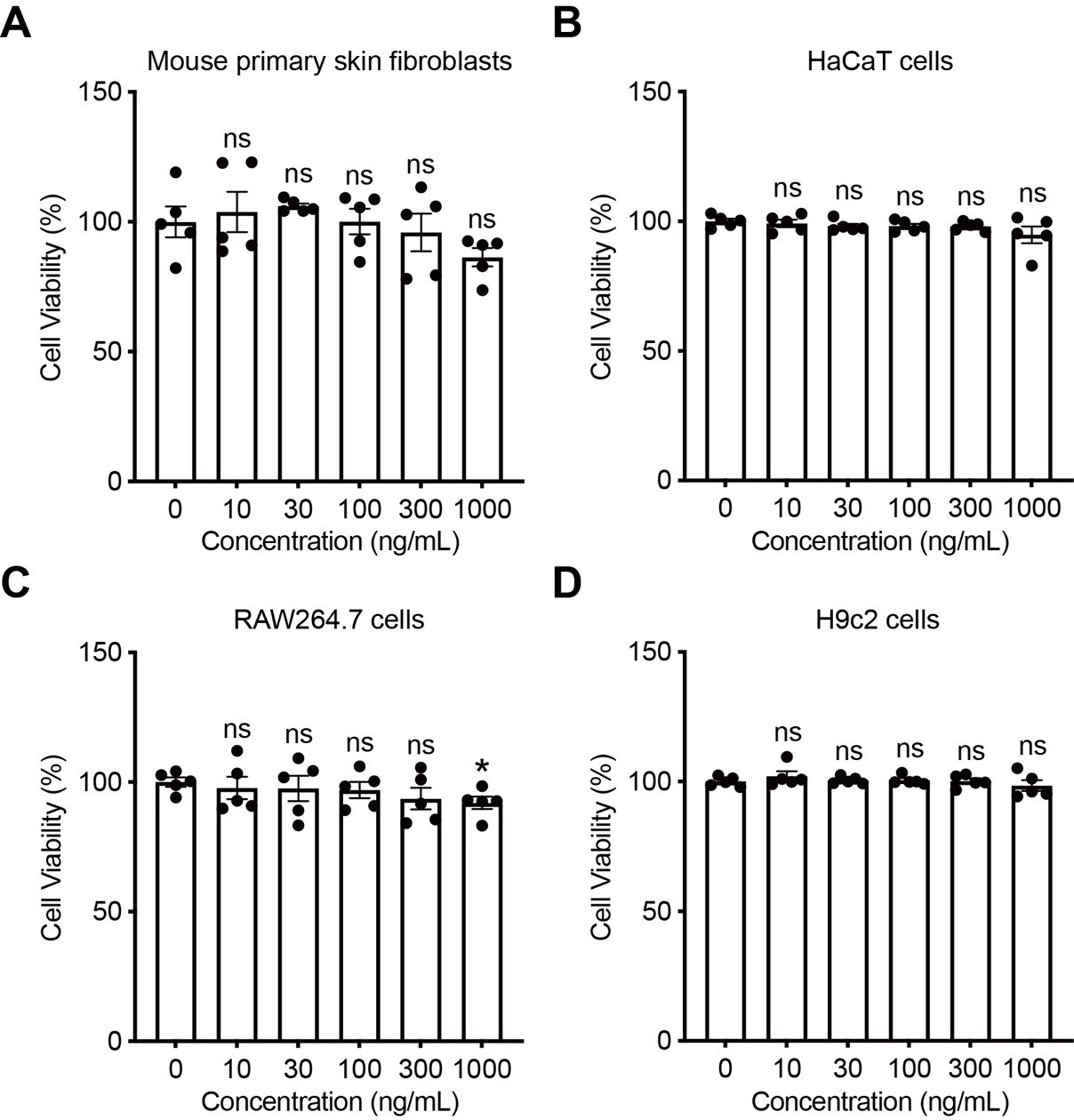


**Figure S8.** Cinobufagin does not inhibit the proliferation of mouse primary skin fibroblasts. Mouse primary skin fibroblasts (A), human keratinocytes HaCaT cells (B), mouse leukemic monocyte/macrophage RAW264.7 cells (C), and rat heart embryonic H9c2 cells (D) were analyzed using CCK8 assay. Data are represented as mean ± SEM, *n* = 5. *P* values are determined by two-tailed unpaired Student’s *t*-test. **P* < 0.05, ns, not significant, indicated group vs. 0 group.


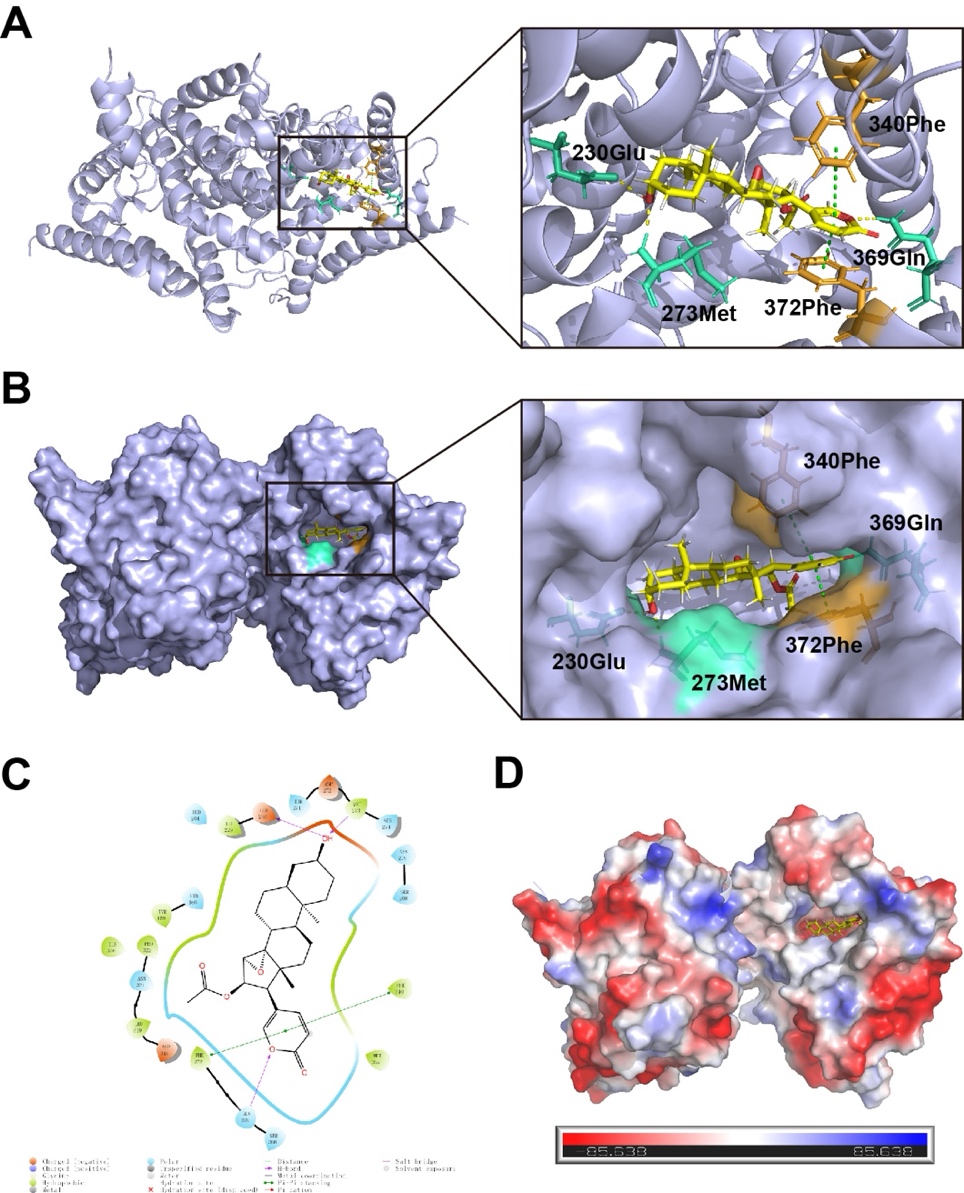


**Figure S9.** Molecular docking simulation experiments show the binding of Cinobufagin to PDE4D. A) Three-dimensional structure of Cinobufagin-PDE4D molecular docking. B) Cinobufagin is located within the molecular pocket of PDE4D protein. C) Two-dimensional structure of Cinobufagin-PDE4D. D) Surface electrostatic forces of Cinobufagin-PDE4D.


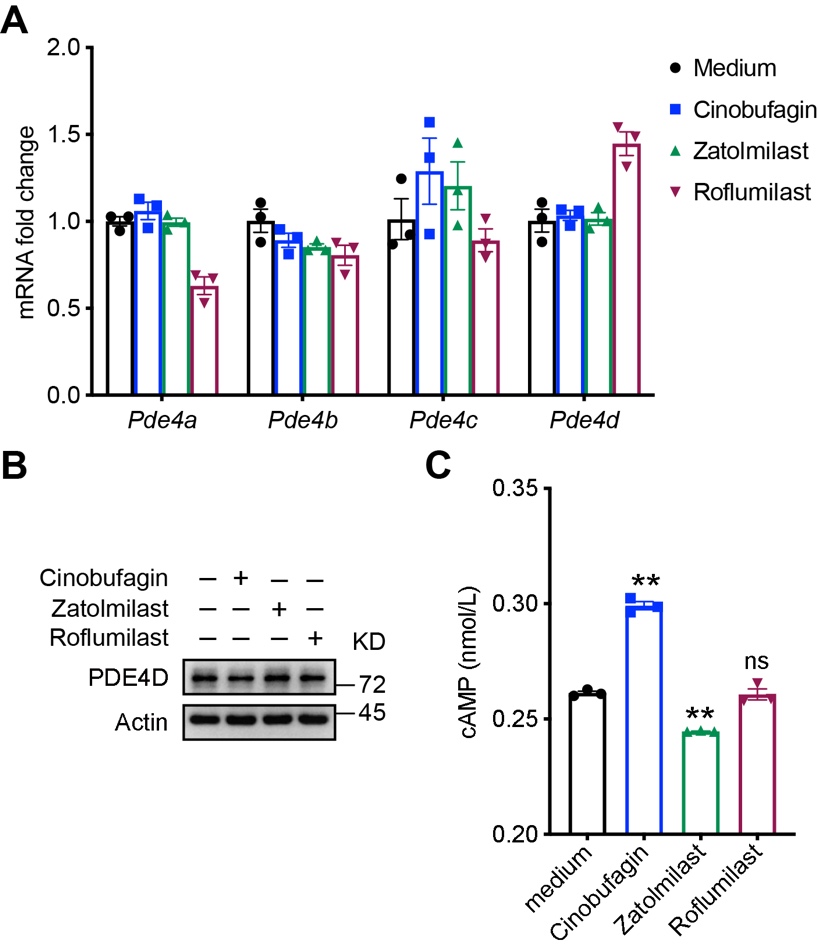


**Figure S10.** PDE4 inhibitors validation. Mouse primary skin fibroblasts derived from C57BL/6 mice were treated with or without PDE4 inhibitors. *Pde4a*, *Pde4b*, *Pde4c*, and *Pde4d* mRNA levels (A), western blot analysis for PDE4D expressions in cell lysates (B), and ELISA for cAMP levels in cell supernatant (C). Data are represented mean ± SEM, *n* = 3. *P* values are determined by two-tailed unpaired Student’s *t*-test for (C). ***P*<0.01, ns, not significant, indicated group vs. medium group.


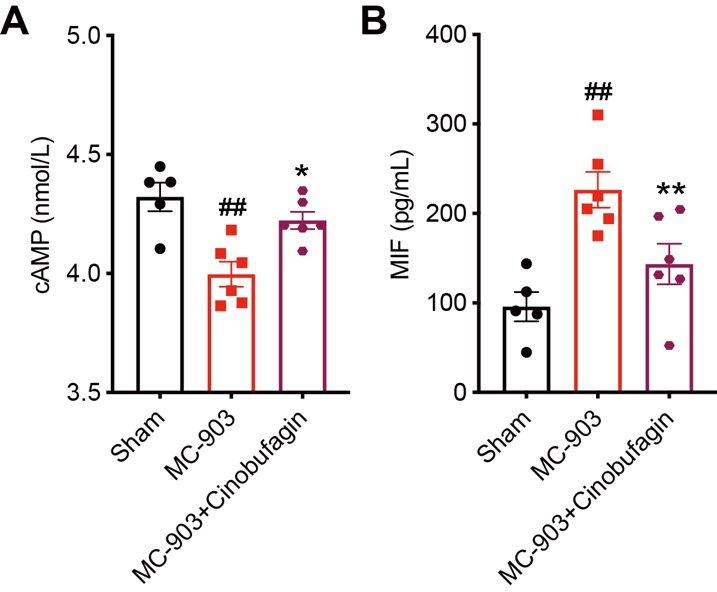


**Figure S11.** Cinobufagin increases cAMP levels and decreases MIF levels. ELISA for cAMP levels (A) and the MIF levels (B) in serum of Sham, MC-903-induced, and Cinobufagin-treated mice. Data are represented as mean ± SEM, *n* = 5-6. The *P*-values are determined by a two-tailed unpaired Student’s *t*-test. ^##^*P* < 0.01, MC-903 group vs. Sham group; **P* < 0.05, ***P* < 0.01, Cinobufagin-treated group vs. MC-903 group.


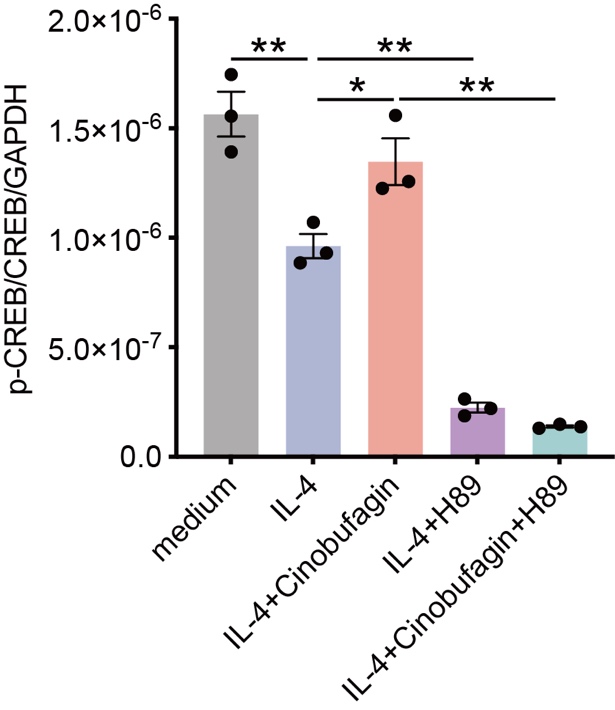


**Figure S12.** Cinobufagin increases the level of phosphorylated CREB. Western blot bands in Figure 7D were quantified in grayscale using image J software, Graphpad Prism 9 plotted the graphs. Data are represented mean ± SEM, *n* = 3. *P* values are determined by two-tailed unpaired Student’s *t*-test. ***P*<0.01, **P*<0.05.


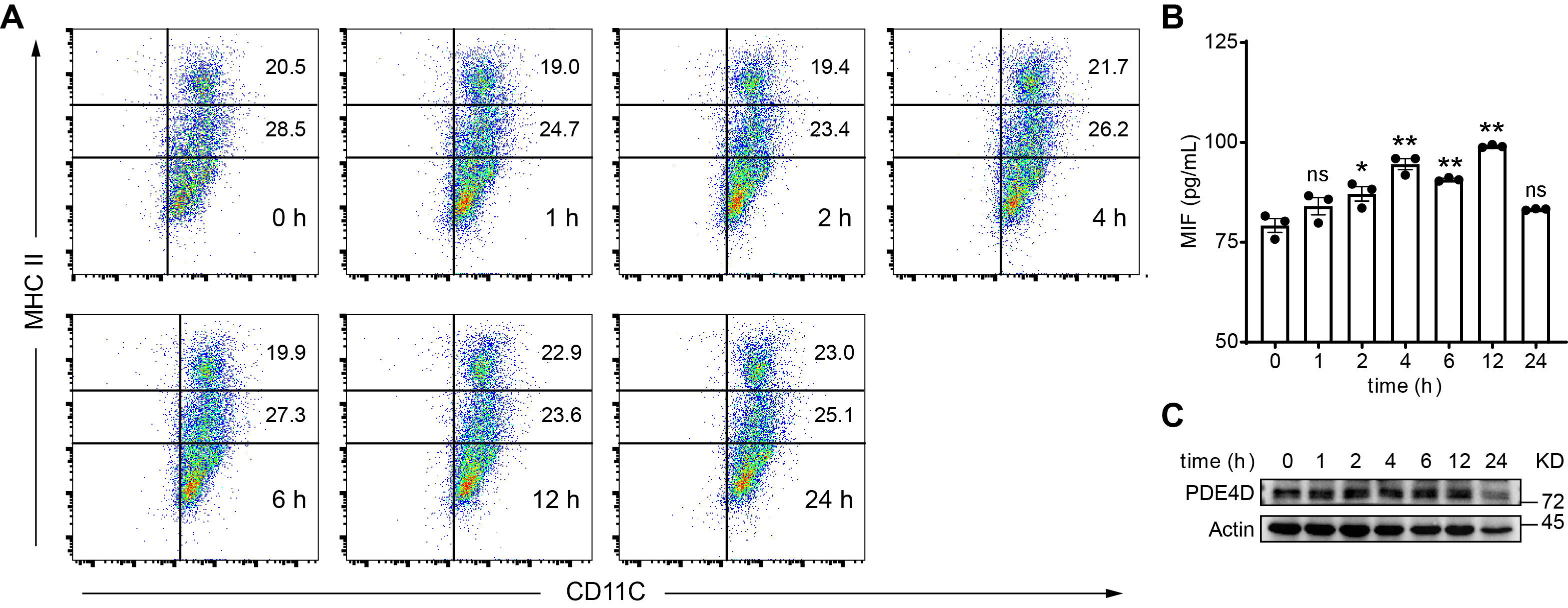


**Figure S13.** IL4-stimulated fibroblasts promote dendritic cell activation. A) Flow cytometry for detecting the expression of CD11C and MHC II on the surface of dendritic cells. B) ELISA for detecting MIF levels in supernatants of fibroblasts co-cultured with dendritic cells for the indicated time. C) Western blot analysis of PDE4D expression level in fibroblasts after co-culturing with dendritic cells for the indicated time. Data are represented as mean ± SEM, *n* = 3. The *P* values are determined by two-tailed Student’s *t*-test for (B). **P* < 0.05, ***P* < 0.01, ns, not significant, indicated group vs. 0 h group.


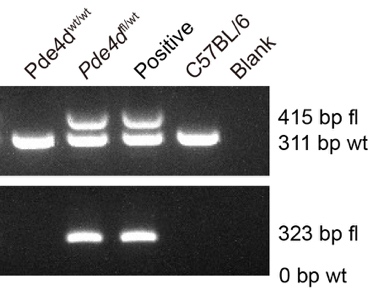


**Figure S14.** Deletion efficiency of PDE4D in *Pde4d*^-/-^ mice. Genotype identification of *Pde4d*^-/-^ mice and wild-type mice tail.


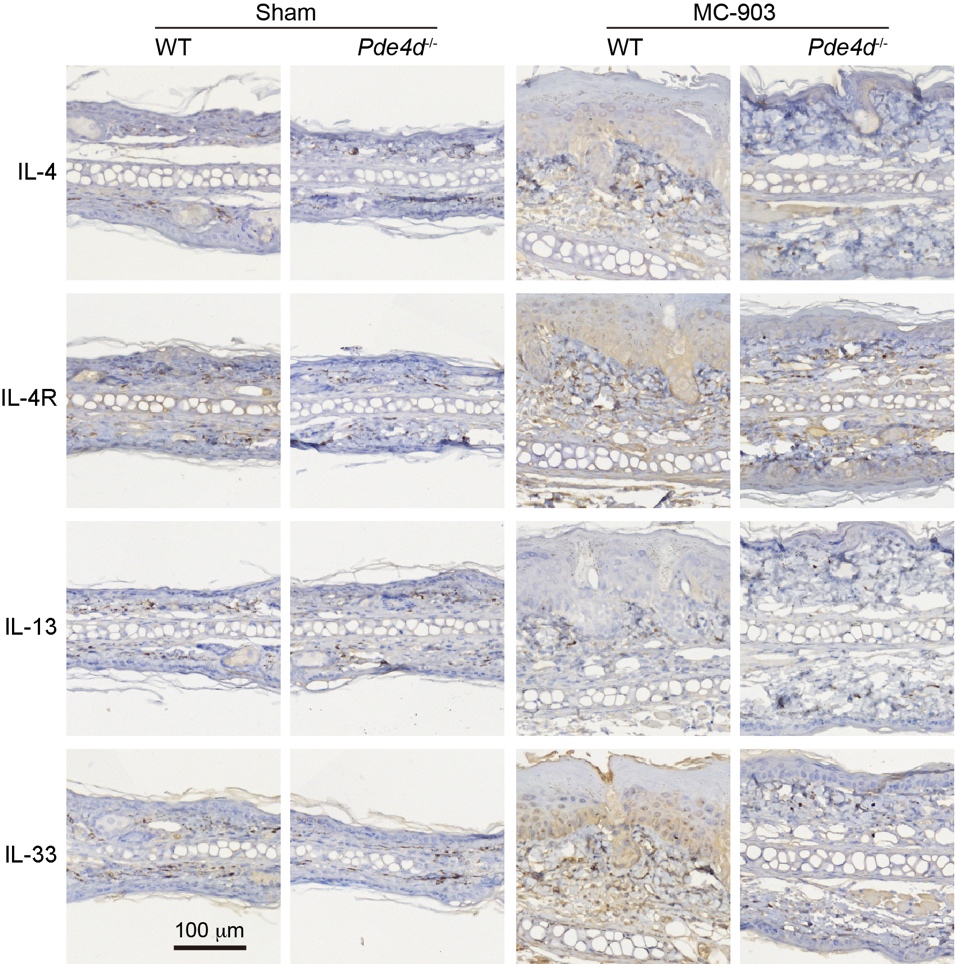


**Figure S15.** PDE4D knockout reduces the expression of Th2-type cytokines in mouse ears. Immunostaining analysis of IL-4, IL-4R, IL-13, and IL-33 protein levels in ear tissues of wild-type and *Pde4d*^-/-^ mice following MC-903 treatment. Scale bar, 100 μm.


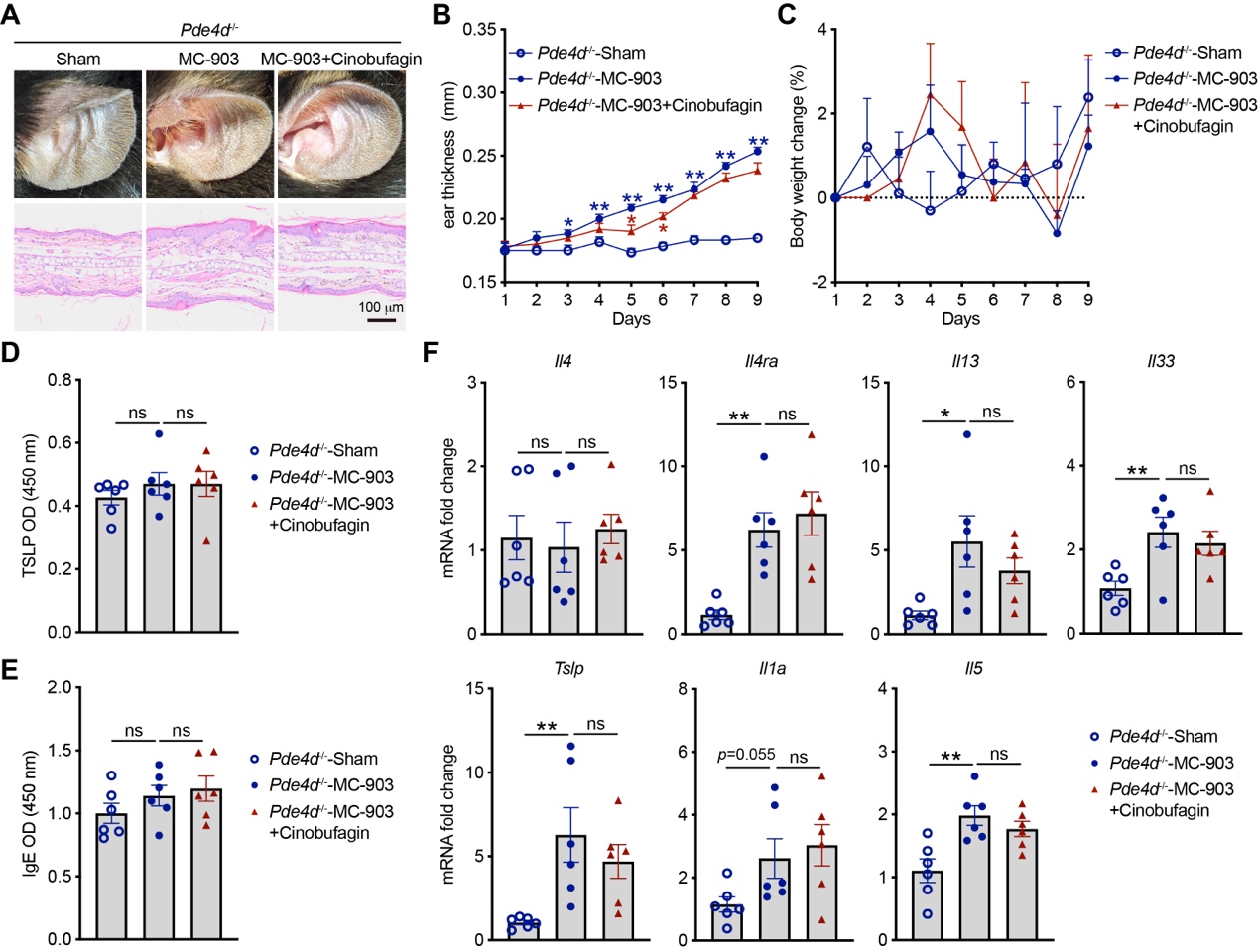


**Figure S16.** Cinobufagin fails to improve morbidity in MC-903-induced PDE4D knockout mice. *Pde4d*^-/-^ male and female mice (*n* =6/group) were orally administered with cinobufagin and treated with MC-903 for nine days. A) Phenotypic representation (*top*) and H&E staining (*bottom*) of mouse ears of the indicated groups. Scale bar, 100 μm. B) Thickness of mouse ears of the indicated groups during the disease process. C) Percent change in body weight of the indicated groups during the disease process. D, E) ELISA quantification of TSLP (D) and IgE (E) levels in mouse serum. F) Quantitative PCR analysis of mRNA encoding Th2 type cytokines in mouse ears. Results were normalized to *Gapdh* expression. Data are represented as mean ± SEM, *n* = 6. *P* values are determined by Tukey’s multiple-comparison test (B), (D)-(F). **P* < 0.05, ***P* < 0.01, ns, not significant.

**Supplementary Table S1.** Potential target protein information of Cinobufagin.

| No | Accession | -lg*P* | Peptides | R | Description |
| --- | --- | --- | --- | --- | --- |
| 1 | PK1IP | 120.21 | 10 | 0.999 | p21-activated protein kinase-interacting protein 1 |
| 2 | KMT2D | 53.05 | 7 | 0.999 | Histone-lysine N-methyltransferase 2D |
| 3 | SRCAP | 51.63 | 6 | 0.995 | Helicase SRCAP |
| 4 | HDGR2 | 114.29 | 11 | 0.989 | Hepatoma-derived growth factor-related protein 2 |
| 5 | DCD | 86.35 | 6 | 0.919 | Dermcidin |
| 6 | DNJA1 | 97.94 | 7 | 0.899 | DnaJ homolog subfamily A member 1 |
| 7 | PDE4D | 58.38 | 5 | 0.889 | cAMP-specific 3' 5'-cyclic phosphodiesterase 4D |
| 8 | GNL1 | 115.29 | 12 | 0.837 | Guanine nucleotide-binding protein-like 1 |
| 9 | HORN | 65.6 | 5 | 0.786 | Hornerin |
| 10 | IF6 | 134.44 | 9 | 0.784 | Eukaryotic translation initiation factor 6 |
| 11 | AIMP1 | 159.56 | 19 | 0.783 | Aminoacyl tRNA synthase complex-interacting multifunctional protein 1 |
| 12 | RS6 | 193.86 | 27 | 0.761 | 40S ribosomal protein S6 |
| 13 | PESC | 162.3 | 30 | 0.719 | Pescadillo homolog |
| 14 | RFC4 | 80.07 | 7 | 0.713 | Replication factor C subunit 4 |
| 15 | PEPD | 37.66 | 3 | 0.602 | Xaa-Pro dipeptidase |
| 16 | CLCA | 33.5 | 2 | 0.596 | Clathrin light chain A |
| 17 | MYO7B | 44.57 | 5 | 0.594 | Unconventional myosin-VIIb |
| 18 | BI2L1 | 65.51 | 7 | 0.551 | Brain-specific angiogenesis inhibitor 1-associated protein 2-like protein 1 |
| 19 | RL24 | 118.61 | 13 | 0.515 | 60S ribosomal protein L24 |
| 20 | SRGP2 | 49.05 | 5 | 0.456 | GTPase-activating protein 2 |
| 21 | MBB1A | 154.96 | 38 | 0.430 | Myb-binding protein 1A |
| 22 | FARP1 | 147.19 | 19 | 0.410 | ARHGEF and pleckstrin domain-containing protein 1 |

**Supplementary Table S2.** Primers for quantitative PCR analysis.

| Primers | Sequences (5′-3′) |
| --- | --- |
| Mouse-*Junb*-F | TCACGACGACTCTTACGCAG |
| Mouse-*Junb*-R | CCTTGAGACCCCGATAGGGA |
| Mouse-*Mif*-F | GGCTCGGTGGTACAGTTCAAG |
| Mouse-*Mif*-R | CCGGAATCGAATCTGCCTCAT |
| Mouse-*Cxcr4*-F | GACTGGCATAGTCGGCAATG |
| Mouse-*Cxcr4*-R | AGAAGGGGAGTGTGATGACAAA |
| Mouse-*Ccl5*-F | GCTGCTTTGCCTACCTCTCC |
| Mouse-*Ccl5*-R | TCGAGTGACAAACACGACTGC |
| Mouse-*Il4a*-F | ACTCCCAAACTCCGAGTCACA |
| Mouse-*Il4a*-R | GGCCCGTCCACTCATGTTC |
| Mouse-*Il4ra*-F | TCTGCATCCCGTTGTTTTGC |
| Mouse-*Il4ra*-R | GCACCTGTGCATCCTGAATG |
| Mouse-*Il13*-F | ACCGAAATGTTGATAGCGACAG |
| Mouse- *Il13*-R | ACAATGCTCTGACAAATGCGTA |
| Mouse-*Il33*-F | TCCAACTCCAAGATTTCCCCG |
| Mouse-*Il33*-R | CATGCAGTAGACATGGCAGAA |
| Mouse-*Tslp*-F | ACGGATGGGGCTAACTTACAA |
| Mouse-*Tslp*-R | AGTCCTCGATTTGCTCGAACT |
| Mouse-*Il1a*-F | CGAAGACTACAGTTCTGCCATT |
| Mouse-*Il1a*-R | GACGTTTCAGAGGTTCTCAGAG |
| Mouse-*Il5*-F | CCCAGGGCGACTGTAACATC |
| Mouse-*Il5*-R | GCAATGTAGATCCTCATGGCAT |
| Mouse-*Gapdh*-F | AGGTCGGTGTGAACGGATTTG |
| Mouse-*Gapdh*-R | TGTAGACCATGTAGTTGAGGTCA |
